# Footprints of local adaptation span hundreds of linked genes in the Atlantic silverside genome

**DOI:** 10.1002/evl3.189

**Published:** 2020-08-19

**Authors:** Aryn P. Wilder, Stephen R. Palumbi, David O. Conover, Nina Overgaard Therkildsen

**Affiliations:** ^1^ Department of Natural Resources Cornell University Ithaca New York 14853; ^2^ Current address: San Diego Zoo Institute for Conservation Research Escondido California 92027; ^3^ Department of Biology, Hopkins Marine Station Stanford University Pacific Grove California 93950; ^4^ Department of Biology University of Oregon Eugene Oregon 97403

**Keywords:** Cline, divergent selection, gene flow, linkage disequilibrium, supergenes

## Abstract

The study of local adaptation in the presence of ongoing gene flow is the study of natural selection in action, revealing the functional genetic diversity most relevant to contemporary pressures. In addition to individual genes, genome‐wide architecture can itself evolve to enable adaptation. Distributed across a steep thermal gradient along the east coast of North America, Atlantic silversides (*Menidia menidia*) exhibit an extraordinary degree of local adaptation in a suite of traits, and the capacity for rapid adaptation from standing genetic variation, but we know little about the patterns of genomic variation across the species range that enable this remarkable adaptability. Here, we use low‐coverage, whole‐transcriptome sequencing of Atlantic silversides sampled along an environmental cline to show marked signatures of divergent selection across a gradient of neutral differentiation. Atlantic silversides sampled across 1371 km of the southern section of its distribution have very low genome‐wide differentiation (median *F*_ST_ = 0.006 across 1.9 million variants), consistent with historical connectivity and observations of recent migrants. Yet almost 14,000 single nucleotide polymorphisms (SNPs) are nearly fixed (*F*_ST_ > 0.95) for alternate alleles. Highly differentiated SNPs cluster into four tight linkage disequilibrium (LD) blocks that span hundreds of genes and several megabases. Variants in these LD blocks are disproportionately nonsynonymous and concentrated in genes enriched for multiple functions related to known adaptations in silversides, including variation in lipid storage, metabolic rate, and spawning behavior. Elevated levels of absolute divergence and demographic modeling suggest selection maintaining divergence across these blocks under gene flow. These findings represent an extreme case of heterogeneity in levels of differentiation across the genome, and highlight how gene flow shapes genomic architecture in continuous populations. Locally adapted alleles may be common features of populations distributed along environmental gradients, and will likely be key to conserving variation to enable future responses to environmental change.

**Impact Statement**

The Atlantic silverside shows a remarkable degree of local adaptation in phenotypic traits, but the genomic basis is unknown. Whole transcriptome sequence data from silversides sampled across their distribution reveal thousands of single nucleotide polymorphisms nearly fixed for opposite alleles across locations, comprising over 1% of variants in the transcriptome, concentrated into large blocks of tightly linked genes with functions related to well‐described adaptations. Absolute divergence was elevated across these genes, indicating the effects of diversifying selection acting on these loci under gene flow, and demographic models that include both migration and selection best fit the transcriptome‐wide data. Because most of the genome shows minimal geographic differentiation due to ongoing gene flow, these massive blocks of fixed differences comprise one of the most striking examples reported to date of how gene flow and selection cluster adaptive variation across the genome. The geographic distribution of variation suggests that protection of populations across environmental gradients may help preserve a reservoir of adaptive potential in the face of human impact.

Local adaptation is a critical generator of ecologically relevant genetic and phenotypic variation. Gene flow can both facilitate and counteract adaptation (Tigano and Friesen 2016). Studying the interplay between the homogenizing effect of gene flow and diversifying effect of selection allows an examination of adaptation to contemporary pressures (Whitlock 2015), and the mechanisms key to shaping the potential response to future environmental shifts (Hoffmann and Sgro 2011). Theoretical models predict that the ability to adapt to local conditions despite ongoing gene flow depends on the genetic basis of adaptive traits and overall levels of genetic variability (Haldane 1930; Yeaman 2015), but we still have a limited understanding of the genomic patterns underlying adaptive divergence in highly connected natural populations, particularly for complex traits with a multigenic basis.

The Atlantic silverside (*Menidia menidia*), a marine fish distributed across a steep thermal gradient along the east coast of North America (Baumann and Doherty 2013), presents a valuable natural system for studying rapid evolution and local adaptation in the face of gene flow. A substantial body of evidence from lab and field studies has demonstrated that this very abundant species (with effective population sizes in the millions; Lou et al. 2018) exhibits a remarkable degree of local adaptation in numerous traits across latitudes (Conover et al. 2009). Among the most widely recognized is north‐south variation in growth rate: short growing seasons in the north are associated with genetically determined faster growth rates, compared to longer growing seasons and slower intrinsic growth rates in the south. These genetic differences counteract environmental influences on growth rates, resulting in similar body sizes despite vast differences in local environmental conditions across the species range (Conover and Present 1990; Arnott et al. 2006). Generally termed counter‐gradient variation, this pattern is also seen in many other fish, frogs, and marine invertebrates (Conover and Schultz 1995; Conover et al. 2009). Counter‐gradient and co‐gradient variation (in which the environment enhances the genetic effect on the phenotype) in myriad other traits have been described in Atlantic silversides, including vertebral number, temperature‐dependent sex determination, swimming performance, lipid storage, and spawning behavior (Conover and Baumann 2009). Common garden experiments have established that these traits are genetically based and can vary over less than 100 km (Conover and Present 1990; Hice et al. 2012).

Importantly, gene flow appears to be high across much of the geographic region where trait divergence is observed. Mitochondrial sequence data define three regional subdivisions of the Atlantic silverside distribution: south of Cape Cod, the Gulf of Maine, and the Gulf of St. Lawrence, with limited substructure within each (Mach et al. 2011; Lou et al. 2018; Fig. 1A). Demographic modeling has suggested that the Gulf of Maine and Gulf of St. Lawrence populations were established from the south after the Last Glacial Maximum, whereas localities extending from Cape Cod to Florida were occupied and connected by ongoing gene flow through the Last Glacial Maximum (Lou et al. 2018).

**Figure 1:**
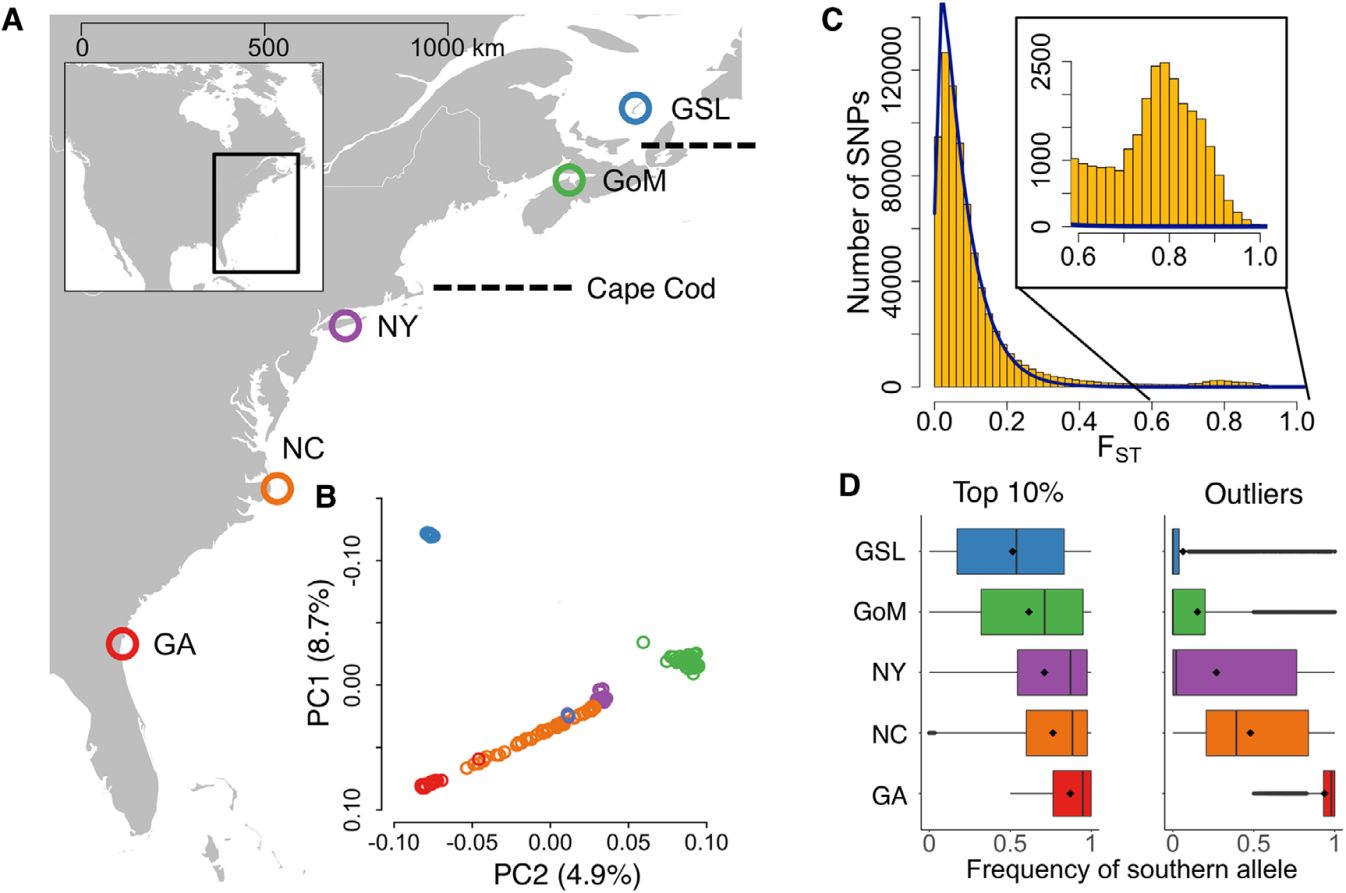
Genetic variation of Atlantic silversides across the east coast of North America. (A) Collection sites in Georgia (GA), North Carolina (NC), New York (NY), Gulf of Maine (GoM), and Gulf of St. Lawrence (GSL). Broken lines indicate boundaries for previously identified phylogeographic regions. (B) Principal components 1 and 2 of transcriptome‐wide variation among individuals, colored by sampling locations in panel A. (C) Transcriptome‐wide distribution of global *F*_ST_ across all populations (yellow bars) fit to a *χ* ^2^ distribution (blue line) expected under neutrality. The inset zooms in on the tail of the distribution. (D) Boxplots of the frequency of the southern alleles at SNPs in the top 10% of the global *F*_ST_ distribution (left panel) and outlier SNPs (right panel).

Under gene flow, strong linkage among co‐adapted loci can preserve adaptive trait complexes that otherwise might be broken apart by recombination (Dobzhansky and Dobzhansky 1970; Kirkpatrick and Barton 2006; Yeaman 2013), a mechanism that has been proposed to underlie adaptation in diverse taxa (Samuk et al. 2017; Wellenreuther and Bernatchez 2018). Studies in the Atlantic silverside have provided evidence of correlations among locally adaptive traits, raising the possibility that suites of co‐adapted alleles associated with a latitudinal gradient may be maintained by genetic linkage (Walsh et al. 2006; Hice et al. 2012; Salinas et al. 2012). In a laboratory experiment, for example, size‐selective harvest of Atlantic silversides led to striking evolution over just four generations, not only in growth rates but also in several correlated traits (Conover and Munch 2002; Walsh et al. 2006). We recently found that this selective response was associated with a dramatic shift in alleles across one cluster of hundreds of genes held together in tight linkage disequilibrium (LD) (Therkildsen et al. 2019). With reference data from wild populations, we also showed that this same genomic block is nearly fixed for opposite alleles across a natural cline in growth rates between the northern and southern extents of the silverside range, suggesting that the rapid multi‐trait response to selection in the experiment was fueled by pre‐existing variation and genomic architecture shaped by diversifying selection across the latitudinal cline (Therkildsen et al. 2019). Yet, this targeted screen revealed only how genomic regions that responded to experimental selection vary among wild silverside populations. It did not explore the broader signatures of natural selection underlying the pronounced local adaptation of multiple traits described in the wild, nor evaluate to what extent such signatures are linked, a feature we may predict to be abundant based on high gene flow in this system.

Here, we investigate for the first time the genome‐wide patterns of variation and genomic architecture that underlie the remarkable adaptive divergence maintained in Atlantic silversides despite high connectivity across latitudes. Using low‐coverage, whole genome sequence data mapped to a reference transcriptome, we characterize genomic differentiation and signatures of selection across the four locations referenced in Therkildsen et al. (2019), plus one intermediate sampling locality near a common biogeographic breakpoint for marine species. We expect that divergent selection under gene flow should enhance both differentiation (*F*_ST_) and absolute divergence (*d_XY_*) of locally adaptive alleles (Cruickshank and Hahn 2014; Delmore et al. 2018), and we predict tighter linkage among adaptive alleles where gene flow is higher (Yeaman and Whitlock 2011; Samuk et al. 2017). Shaped by a latitudinal gradient and steep thermal cline, genetic variation in this species has allowed for pronounced spatial and temporal adaptive responses, and may serve as the foundation for future adaptive shifts in the face of climate change.

## Methods

### LIBRARY PREPARATION AND BIOINFORMATICS

Adult silversides were collected during the spring spawning period between 2005 and 2007 from five locations: Jekyll Island, Georgia (GA; *n* = 48), Oregon Inlet, North Carolina (NC; *n* = 47), Patchogue, New York (NY; *n* = 49), Minas Basin, Nova Scotia in the Gulf of Maine (GoM; *n =* 50), and Magdalen Island, Quebec in the Gulf of St. Lawrence (GSL; *n* = 42; Fig. 1A). Our first round of sequencing included all individuals from four of these locations: GA, NY, GoM, and GSL. A separate barcoded genomic library was prepared for each individual as described in Therkildsen and Palumbi (2017). The libraries were pooled in equimolar amounts and sequenced using paired‐end 125 bp reads on an Illumina HiSeq instrument, randomly distributing sample libraries across lanes. Whole‐genome sequence reads were mapped to an Atlantic silverside reference transcriptome (Therkildsen and Baumann 2020) to a final average depth of 1.5×. Because several clusters of single nucleotide polymorphisms (SNPs) showed fixation for either the reference or alternate allele in each of our four original populations (details below), precluding robust analysis of LD, we added a second round of sequencing of samples from an intermediate location, NC, located near Cape Hatteras, a region of steep thermal change and a phylogeographic break for a number of marine species (Briggs and Bowen 2012; Pappalardo et al. 2015). For these samples, individually barcoded genomic libraries were prepared with a similar protocol and sequenced using paired‐end 75 bp reads (see Methods in the Supporting Information for details), and mapped using the same parameters as for the other samples.

We provide a concise overview of each step in our analysis here; additional details are provided in the Methods section of the Supporting Information. To avoid calling spurious variants due to differences in read length (Leigh et al. 2018), biallelic SNPs were called across the four original populations (GA, NY, GoM, and GSL; *n* = 189) in the program ANGSD version 0.912 (Korneliussen et al. 2014), and downstream analyses were performed using this set of SNPs for all five populations. The filtered dataset comprised 1,904,119 biallelic SNPs across the ∼52 MB transcriptome. Contigs had a mean of 92.57 SNPs and an average of ∼1 SNP per 24 bp (the average contig length was 2537bp), and high overall nucleotide diversity within all populations (Table S1). Except where noted, all downstream analyses were based on genotype likelihoods to incorporate uncertainty in genotyping.

### POPULATION GENOMIC ANALYSIS

Major and minor alleles, minor allele frequencies (MAF), and *F*_ST_ were estimated in ANGSD from genotype likelihoods for each SNP with data for at least 10 individuals per population (Korneliussen et al. 2014). To identify *F*_ST_ outlier SNPs in the transcriptome, we compared the global *F*_ST_ (estimated from MAF output by ANGSD) to an expected *χ* ^2^ distribution fit to ∼33,000 SNPs (randomly sampled and pruned for LD), trimming 5% from each side of the *F*_ST_ distribution in OutFLANK (Whitlock and Lotterhos 2015). We then applied the *χ* ^2^ fit to all SNPs across the transcriptome to identify *F*_ST_ outliers using a false discovery rate of *q* < 0.05. Absolute divergence (*d_XY_*) for each contig was estimated at all SNPs from MAF output by ANGSD using the CalcDxy.R script in ngsTools (Fumagalli et al. 2014), accounting for the length of each contig. Principal components analysis (PCA) was performed by computing eigenvectors in R from the covariance matrix between individuals estimated in PCAngsd (Meisner and Albrechtsen 2018). We also performed admixture analysis using PCAngsd, which estimates the optimal number of clusters (*K*) and the posterior assignment probabilities of individuals to each cluster. We estimated interpopulation ancestry between NY and GSL, and between GA and NY from a subset of 100,000 SNPs under *K* = 4 in the program *entropy* (Gompert et al. 2014). To evaluate whether low genome‐wide differentiation reflects connectivity through gene flow, we used the Moments pipeline (Jouganous et al. 2017; Leaché et al. 2019) to fit and compare simple demographic models to the two‐dimensional site frequency spectrum (2dSFS) between GA and NY. One SNP per contig was randomly selected to generate the empirical 2dSFS, and competing models were ranked by Akaike information criterion (AIC). We compared eight evolutionary models for the two populations with the following migration scenarios: (1) no migration, (2) symmetrical migration, and (3) secondary contact. We extended each of these three scenarios in three models allowing for reduced Ne across a proportion of the genome due to background/positive selection, and extended the two gene flow scenarios in two models allowing for decreased migration across a proportion of the genome due to, for example, barrier loci (Rougeux et al. 2017).

Within each intermediate latitude population (NC, NY, and GoM), we estimated LD between all pairs of *F*_ST_ outlier SNPs (identified in the OutFLANK analysis above) using default settings in the program ngsLD (Fox et al. 2019). LD analysis within GSL and GA was not meaningful because of the near fixation for one allele at almost all outlier SNPs. We used the *D*′ statistic output by ngsLD and filtered out SNPs with MAF <0.1. To define blocks of LD, we first estimated the mean *D*′ value between pairs of contigs with more than two outlier SNPs and then performed hierarchical clustering of pairwise mean LD between contigs in each population in the R package *hclust*. We anchored contigs from the silverside transcriptome onto the genome of a related species, the medaka (*Oryzias latipes*), to approximate the relative positions of silverside contigs. Comparisons of fish genomes tend to show few interchromosomal rearrangements (Amores et al. 2014; Rondeau et al. 2014; Pettersson et al. 2019), and thus we expect to sort contigs correctly to chromosomes, but we do not expect to infer the location of a contig within a chromosome. Although we leveraged the medaka genome to infer the likelihood that LD clusters are physically linked on the same chromosome, all analyses of LD blocks were based on hierarchical clustering of silverside contigs without using any genomic information from medaka. To visualize haplotype patterns within LD blocks, we created heatmaps showing the most likely genotypes (inferred from genotype likelihoods for each individual at outlier SNPs), defining the “southern” allele as the major allele in GA.

We used snpEff to predict the functional effects of SNPs (Cingolani et al. 2012), and tested for enrichment of variant types in the top 1% tail of the *F*_ST_ distribution, and among SNPs in LD blocks, relative to their proportions across the rest of the transcriptome using a *χ* ^2^ goodness‐of‐fit test. Contigs were annotated with gene functions based on sequence similarity to reference fish species. We tested for enrichment of Biological Processes GO terms in each LD block by scoring each gene with the sum of outlier SNPs, and tested significance using the gene score resampling (GSR) method in the program *ErmineJ* (Gillis et al. 2010).

## Results and Discussion

### NEAR FIXATION OF OPPOSITE ALLELES ACROSS HUNDREDS OF GENES DESPITE LOW GENOME‐WIDE DIFFERENTIATION

All five populations formed distinct clusters along principal components of genome‐wide variation (Fig. 1B), but the overall level of differentiation was relatively low (median *F*_ST_ range 0.006‐0.036 between neighboring populations). Set within this background of low genome‐wide differentiation, 32,384 of the ∼1.9 M total SNPs (1.70%) showed greater differentiation than expected from a neutral *χ* ^2^ distribution (false discovery rate *q* < 0.05 in OutFLANK; Fig. 1C), pointing to nonneutral evolutionary forces acting on the genome. Although most differentiated SNPs follow a cline in allele frequencies across latitudes, the extreme *F*_ST_ outlier SNPs (*q* < 0.05) mostly differentiate GA from all the populations to the north (Fig. 1D).

**Figure 2:**
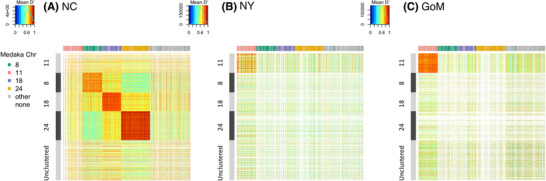
Linkage disequilibrium (LD) between pairs of contigs with outlier SNPs in North Carolina (NC), New York (NY), and Gulf of Maine (GoM). Pixels are colored by mean *D*′ across outlier SNPs in each contig pair. (White pixels show contigs with fewer than two outlier SNPs with MAF > 0.1). Hierarchical clustering of LD between contig pairs clusters all these contigs into four LD blocks (see Fig. S1), indicated by row panels in the symmetrical matrix. Column panels indicate the chromosome each contig maps to in the medaka genome, showing large proportions of contigs in LD blocks mapping to a single medaka chromosome. GA and GSL are not shown because the near fixation of alleles in these populations limits the power of LD analysis.

The majority of the 32,384 outlier SNPs showed strong LD patterns in populations at the center of the geographic range where allele frequencies are intermediate. Hierarchical clustering of LD across contigs revealed that 1171 out of 1710 contigs with extreme outlier SNPs cluster into one of four distinct blocks of high LD (Fig. S1). Anchoring contigs along the medaka (*Oryzias latipes*) genome indicate that the contigs in the four LD blocks predominantly map to four different medaka chromosomes (Fig. 2). Three large blocks (comprising 263, 255, and 391 contigs, respectively) that are polymorphic in the NC population correspond to medaka chromosome (Chr) 8, 18, and 24 (89.5%, 82.3%, and 91.2% of mappable contigs map to these chromosomes, respectively; Fig. 2A). In GoM, another distinct LD block of 263 contigs maps mostly to medaka Chr 11 (72.9% of mappable contigs; Fig. 2C).

**Figure 3:**
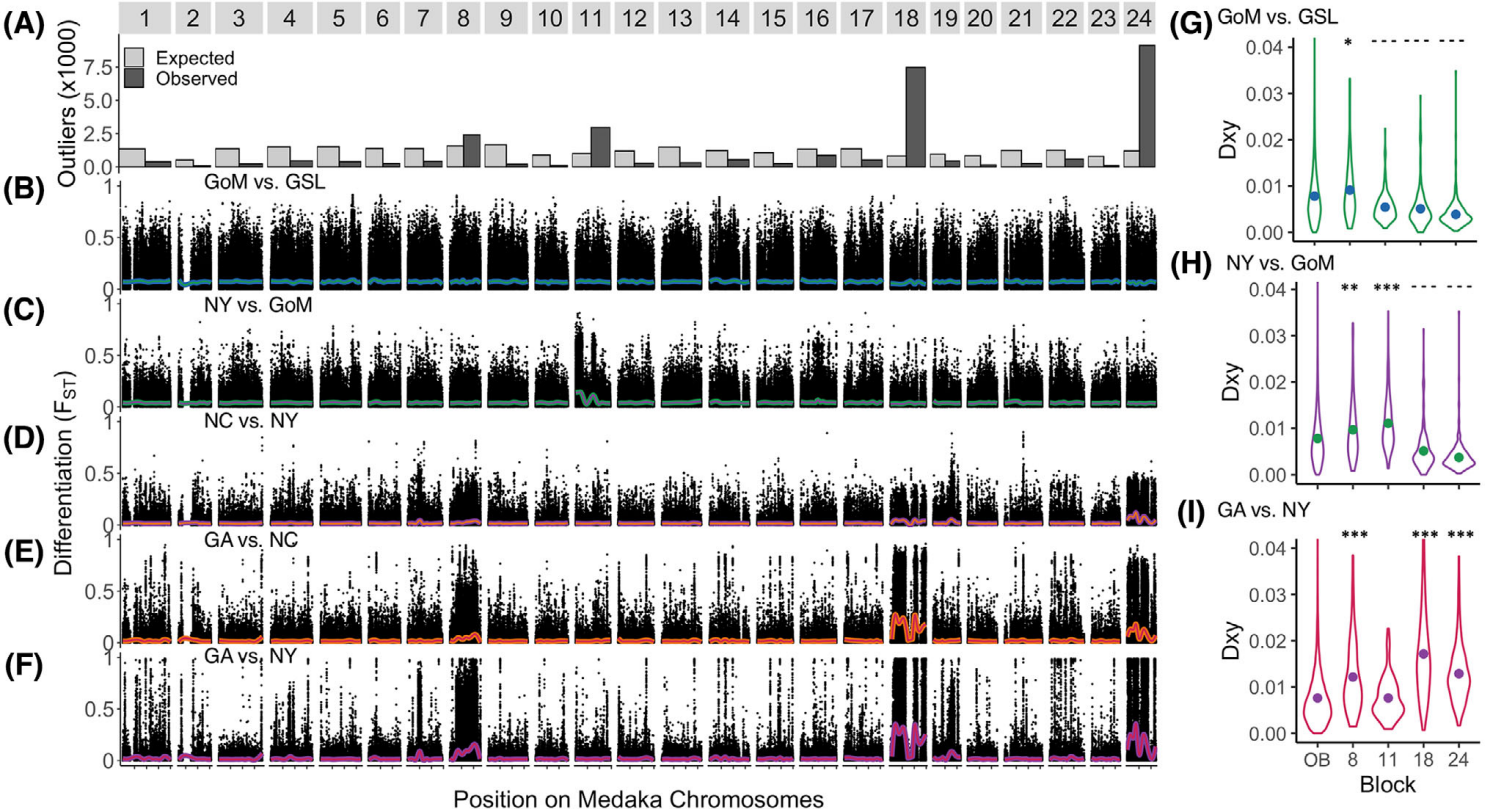
Variation among pairs of Atlantic silverside populations across contigs anchored to the medaka genome, showing overrepresentation of differentiated SNPs on the four medaka chromosomes corresponding to LD blocks in Figure 2. Few interchromosomal rearrangements between fish genomes suggest that contigs sort correctly to chromosomes, but we do not expect to robustly infer the location of a contig within a chromosome. (A) Observed versus expected number of global *F*_ST_ outlier SNPs on each medaka chromosome. (B‐F) *F*_ST_ at individual SNPs for (B) GoM versus GSL, (C) NY versus GoM, (D) NC versus NY, (E) GA versus NC, and (F) GA versus NY, with colored lines showing sliding mean *F*_ST_. (G‐I) Comparison of the distribution of *d_XY_* between population pairs for contigs in LD blocks 8, 11, 18, and 24 and contigs outside blocks (OB). Asterisks above violin plots indicate significantly elevated *d_xy_* in blocks relative to OB; dashes indicate significantly reduced *d_xy_* relative to OB. ^***/‐‐‐^ *P* < 1× 10^–4^; ^**^ *^/‐‐^ P* < 1× 10^–3^; ^*/‐^ *P* < 0.05.

The membership of genes in LD blocks (hereafter referred to as LD blocks 8, 11, 18, and 24) was defined without any a priori information about chromosomal locations in the medaka genome, and thus the high degree of co‐localization on single medaka chromosomes suggests that the contigs within each LD block are likely to be physically linked in the Atlantic silverside genome. Silversides and medaka diverged ∼75 million years ago (Near et al. 2012), but the species share the same chromosome number, 2*n* = 48 (Warkentine et al. 1987), and comparisons of genomes both within the Atherinomorpha clade (to which both species belong; Amores et al. 2014; Miller et al. 2019) and fishes more broadly (Rondeau et al. 2014; Pettersson et al. 2019), tend to show few interchromosomal rearrangements. Thus, although potential intrachromosomal rearrangements limit our ability to infer the order of genes within chromosomes, the nonrandom clustering of outlier SNPs on medaka chromosomes (Fig. 3A), and strong LD among silverside contigs mapping to the same medaka chromosomes (but lower LD among those mapping to different chromosomes; Fig. 2), suggests that the medaka genome provides a reasonable approximation of the genomic location of our contigs.

Pairwise comparisons show that the most striking heterogeneity in genomic differentiation is found within the southernmost phylogeographic region (Lou et al. 2018), between GA and NY (Fig. 3F). Separated by ∼1500 km of coastline, the three populations sampled in this region had the lowest baseline levels of differentiation (genome‐wide median *F*_ST_ = 0.009, 0.007, and 0.006 between GA and NC, NC and NY, and GA and NY, respectively), yet 13,734 SNPs were fixed or nearly fixed (*F*_ST_ > 0.95) for opposite alleles between GA and NY. SNPs in the top 1% of the *F_ST_* distribution were disproportionately nonsynonymous (NS) variants (29.1% more NS variants than the rest of the transcriptome; *χ* ^2^ goodness of fit test; *P* < 2.2 × 10^–16^; Table S2), and the vast majority (99.1%) of them clustered into LD blocks 8, 18, and 24 (with 82.0% mapping to medaka chromosomes 8, 18, and 24, Fig. 3A). There was a clear north‐south split of alleles at these blocks across the range; the three northern populations share the NY allele at almost all outlier SNPs, whereas the opposite alleles are found almost exclusively in GA. In NC, near Cape Hatteras, a region of steep thermal transition, northern and southern alleles on blocks 8, 18, and 24 were at intermediate frequencies (Figs. 3D and 3E).

Genes in the three LD blocks show elevated levels of absolute divergence (*d_XY_*) between GA and NY compared to the rest of the genome (Fig. 3I). This pattern is consistent with a model of differential gene flow where selection promotes divergence at locally adaptive loci, whereas gene flow homogenizes the rest of the genome (Nachman and Payseur 2012; Cruickshank and Hahn 2014). By contrast, among populations where alleles at these LD blocks are shared (i.e., NY, GoM, and GSL), *d_XY_* across these loci is reduced relative to genome‐wide levels (Fig. 3G‐H), indicating linked selection on a shared allele in both populations or in an ancestral population (Irwin et al. 2016; Delmore et al. 2018).

The LD blocks are also enriched for functional variants relative to the rest of the transcriptome, consistent with selection on functional genetic variation (Table S3). LD block 18 had 35.1% more NS variants and LD block 24 had 16.4% more NS variants than expected from transcriptome‐wide proportions (*χ* ^2^ goodness‐of‐fit, both *P* < 2.2 × 10^–16^; Table S3). Although LD block 8 did not show significant enrichment of NS variants, it had 9.0% more UTR variants (*χ* ^2^ goodness‐of‐fit, *P* < 2.2 × 10^–16^; Table S3). The extreme degree of differentiation, enrichment of functional variation, and elevated levels of absolute divergence across the large LD blocks exceed expectations under neutral evolution (Figs. 1C and 3G‐I), and support a role for selection in shaping them.

### EVIDENCE OF SUPPRESSED RECOMBINATION WITHIN LARGE LD BLOCKS

**Figure 4:**
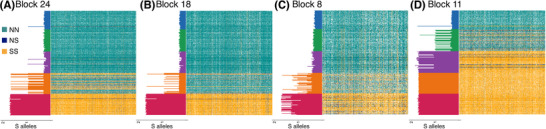
Distribution of genotypes across LD blocks. Genotype likelihoods for silverside individuals (rows) at each outlier SNP (columns) in (A) LD block 24, (B) LD block 18, (C) LD block 8, and (D) LD block 11. Barplots (left) show the average genotype (number of southern alleles) across SNPs for each individual, colored by sampling origin (red: GA, *n* = 48; orange: NC, *n* = 47; purple: NY, *n* = 49; green: GoM, *n* = 50; blue: GSL, *n* = 42). Because of low read depth, heterozygotes appear as mosaics of each of the three possible genotypes (NN, NS, and SS) and have an average number of southern alleles of ∼1 across SNPs.

In the three blocks that are polymorphic along the southern coastline, we quantified genotype likelihoods at outlier SNPs to evaluate the distribution of alleles among individuals. In LD blocks 18 and 24, homozygous individuals had either the northern or southern allele (but not both) at virtually all SNPs, but because the sequencing at ∼1× coverage captures only one allele on average, heterozygous genotypes were a mosaic of the two alleles. In GA and NY, virtually all copies of locally rare alleles at these SNPs were present in the same few individuals (Figs. 4A and B). There was a cline in frequencies of northern and southern haplotypes of both blocks across the three southern populations, with few observations of heterozygotes outside of NC. In LD block 24, four individuals in three populations outside of NC carried both haplotypes (Fig. 4A), and in LD block 18, there was one heterozygote in GA (of *n* = 48 individuals), and no southern haplotypes north of NC (Fig. 4B). Block 8 showed looser associations of alleles across SNPs, but the distinct north‐south haplotype structure was largely maintained, and we observed no southern haplotypes in the three northern populations (Fig. 4C). The fact that heterozygous individuals appear to be heterozygous across the entire blocks, and homozygous individuals are homozygous across them, suggests very strong linkage of northern and southern alleles (respectively) across extended haplotypes.

Long haplotypes in almost perfect LD within populations imply that divergent alleles have limited recombination. Such large swaths (spanning hundreds of genes) of maintained LD point to inversions or other mechanisms of recombination suppression, which may maintain linked genes as stable polymorphisms. Inversions that capture multiple locally adapted alleles can have a selective advantage, and are particularly important when gene flow is high (Noor et al. 2001; Kirkpatrick and Barton 2006). An alternative explanation for high LD is recent admixture of differentiated alleles, but LD decays very rapidly when recombination is unrestricted (Hartl et al. 1997). Yet in NC where frequencies of northern and southern alleles were intermediate and heterozygosity was high (providing plenty of opportunity for recombination), the distinct haplotypes are maintained, suggesting suppressed recombination is maintaining LD across these haplotypes. This conclusion is further corroborated by our previous report of a strong response to size selection on LD block 24 in a laboratory experiment (Therkildsen et al. 2019; the other LD blocks were not identified in the experiment). Established from silversides collected near NY, the initially rare southern haplotype showed a dramatic increase in frequency under strong selection, yet did not recombine with the northern haplotype over 10 generations despite high heterozygosity in the experimental population.

Recombination suppression (e.g., within inversions) may lead to the accumulation of deleterious mutations, providing an alternate explanation for NS variant enrichment seen across these blocks. But because most individuals were homozygous for northern or southern alleles (Fig. 4), recombination within inversion types can facilitate purging of these mutations (Otto and Lenormand 2002; Kirkpatrick 2010), making this explanation less plausible than one of divergent selection on adaptive variation. Although having a contiguous genome will elucidate the genomic mechanisms underlying the recombination suppression revealed in these analyses, the transcriptome data make a clear case for strong selection on suites of genes in LD.

### ATLANTIC SILVERSIDES AS AN EXTREME EXAMPLE OF THE GENOMIC PATTERNS UNDERLYING LOCAL ADAPTATION

The exact width of the highly linked blocks of divergence in the Atlantic silverside genome remains to be determined, but alignment to the medaka genome suggests that they could span much of the four chromosomes on which they are found (Fig. 3). The total length of the 908 contigs that make up the three LD blocks (8, 18, and 24), which are highly differentiated across the southern region between GA and NY, amount to ∼2.88 MB in length. However, the reference contigs exclude introns and intergenic regions that intersperse the transcriptome sequence, indicating that these footprints must span multiple megabases of the Atlantic silverside genome.

Localized genomic regions of elevated intraspecific differentiation have now been identified in numerous species, but often the stretches of elevated differentiation span smaller genomic regions, as is the case for the stickleback (*Gasterosteus aculeatus*; Jones et al. 2012) and African honeybee (Wallberg et al. 2017). In other species, such as the Atlantic cod (*Gadus morhua*), Atlantic herring (*Clupea harengus*), and maize (*Zea mays*), Mb‐scale inversions are under strong divergent selection, but rarely approach fixation of opposite haplotypes between populations (Fang et al. 2012; Pettersson et al. 2019; Kess et al. 2020). The genomic breadth and magnitude of allele frequency differentiation (i.e., near fixation of opposite alleles across Mb‐scale regions against an almost complete absence of genome‐wide differentiation, as seen in the southern part of the range) represents, to our knowledge, one of the most extreme examples reported to date of the degree to which differentiation of locally adapted populations can vary across the genome (compare to other notable examples in Table 1). The increasing availability of genome‐wide SNP data for many species will make the identification of clusters of differentiated alleles easier, and determine whether adaptation to large‐scale environmental differentiation is regularly associated with extreme differentiation across gene clusters.

**Table 1:** Examples of intraspecific differentiation generated by local adaptation in the face of gene flow across populations

Species	Differentiation driven by selection	Size of differentiated region	Adaptation	Genome‐wide differentiation	References
*Zea mays*	AF range ∼0.2‐0.9 across altitudinal cline	∼50 MB inversion (700 genes)	Associated with environmental variables and phenotypic traits	Isolation by distance	Fang et al. 2012
*Clupea harengus*	Max *F*_ST_ ∼0.9	7.2 MB	Salinities and spawning times	Median *F*_ST_ = 0.032	Barrio et al. 2016; Pettersson et al. 2019
*Gadus morhua*	Max *F*_ST_ of SNPs within inversions ∼0.7‐0.8	Several MB, ∼4% of genome	Salinity, temperature, and migration ecotypes	Neutral *F*_ST_ = 0.0012	Hemmer‐Hansen et al. 2013; Kess et al. 2020
*Drosophila melanogaster*	AF range ∼0.2‐1.0 across latitudinal cline	∼5‐15 MB inversion	Size, development, fecundity, other traits	*F*_ST_ ∼0.027‐0.044	Fabian et al. 2012; Kapun et al. 2016
*Apis mellifera* subspecies	Two putative inversions, *F*_ST_ = 0.7 and 0.8	100s of Kb	Highland habitat	*F*_ST_ = 0.05‐0.07	Wallberg et al. 2017
*Menidia menidia*	*F*_ST_ > 0.95 for 13,734 SNPs	Unknown, likely megabases	GO terms related to known adaptations (e.g. lipid storage, metabolism, spawning)	Median *F*_ST_ = 0.006 (GA vs. NY)	This study

### MULTIPLE ADAPTIVE CLINES ACROSS A GRADIENT OF GENOME‐WIDE POPULATION DIFFERENTIATION

In the middle of the silverside range between NY and GoM, genome‐wide differentiation was intermediate (median *F*_ST_ = 0.024), but there were no SNPs fixed for opposite alleles (max *F*_ST_ = 0.93). The most highly differentiated SNPs fell into contigs that formed a tight LD block corresponding to medaka Chr 11 (Fig. 2C). Genes within LD block 11 (spanning 263 contigs that cumulatively encompass 0.70 MB of the transcriptome) had significantly elevated levels of *d_XY_* between NY and GoM (Fig. 3H), and 5.8% more NS variants than the rest of the transcriptome (*χ* ^2^ goodness‐of‐fit, *P* < 1 × 10^–3^; Table S3), but unlike the three LD blocks that distinguish the southern population (GA) from the northern, alleles of LD block 11 transition at a higher latitude, with the southern haplotype predominant in the three southern populations, and the northern haplotype predominant in the North. The different geographic breakpoints among the LD blocks on different chromosomes suggest that they are driven by separate selection pressures across latitudes, consistent with multiple, distinct latitudinal clines for different phenotypic traits in Atlantic silversides (Conover et al. 2009; Hice et al. 2012).

In contrast to the highly connected southern coastline, the two most northerly samples, GoM and GSL, had the highest background level of differentiation among neighboring locations (median *F*_ST_ = 0.036), reflecting the history of independent colonization of GoM and GSL from the southern coastline (Lou et al. 2018). Despite the higher level of background differentiation, no SNP was fixed for opposite alleles (max *F*_ST_ = 0.91), and there was little obvious clustering of highly differentiated SNPs on medaka chromosomes (Fig. 3B).

### LONG‐TERM AND CONTEMPORARY MIGRATION AND GENE FLOW

**Figure 5:**
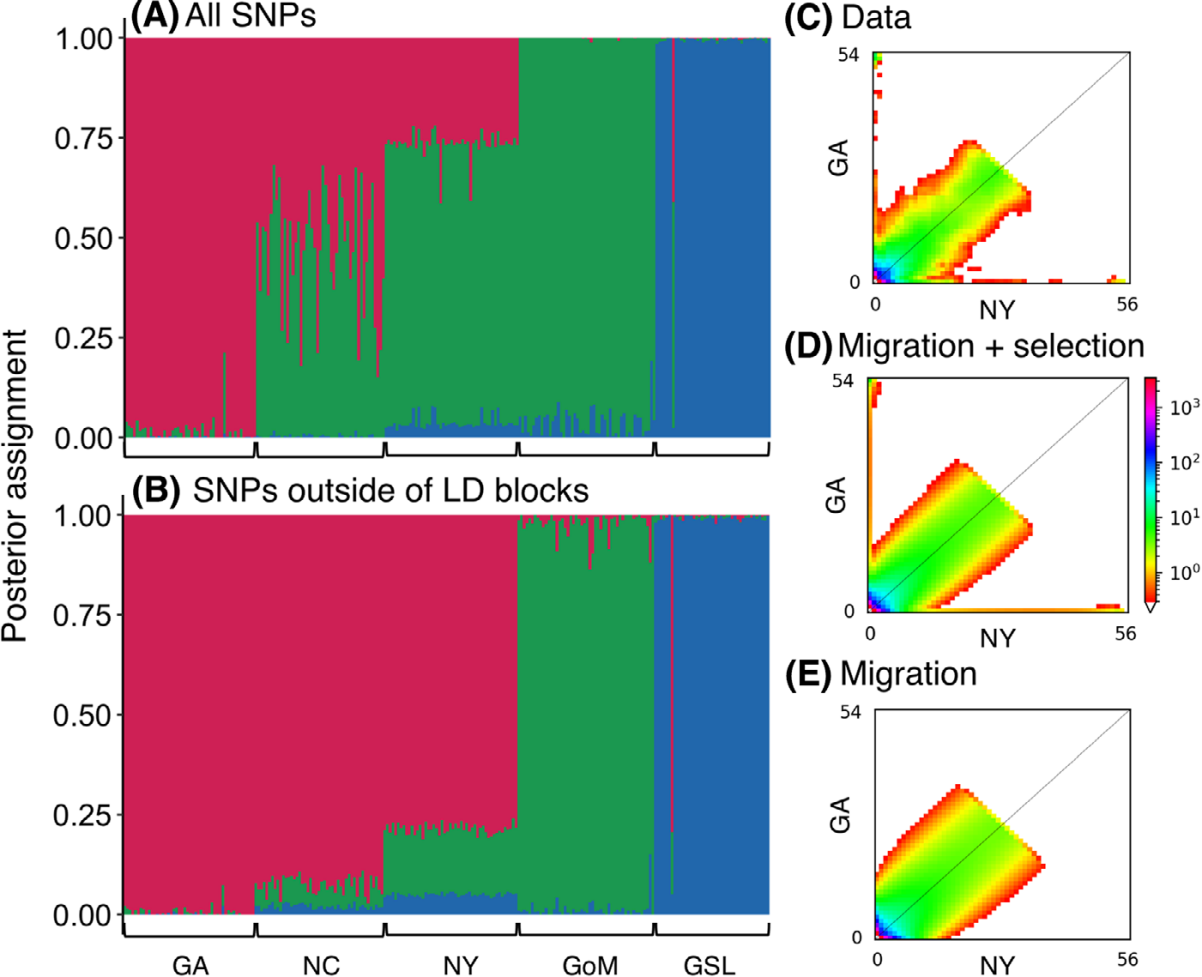
Admixture analysis under an optimally fitted model of *K* = 3 populations shows a cline in posterior assignment of individuals from GA, NC, and NY using all genome‐wide SNPs (A), but shows high assignment of individuals from these locations to one cluster using only SNPs outside LD blocks (B). Observed 2dSFS for GA and NY (C) showed the best fit to a model of symmetrical migration with reduced *N* *_e_* at a subset of loci due to selection (D), compared to other candidate models such as symmetrical migration without selection (E), and those of divergence in isolation without migration. Models that included the effects of selection were able to capture the highly differentiated alleles along the edges of the 2dSFS (D), whereas models that did not include heterogeneity in the genome underpredicted the frequency of these sites in the 2dSFS (E).

Admixture analysis of all SNPs indicate the genomic data best fit a model of *K* = 3 populations, with a gradient in posterior assignment to populations 1 and 2 of individuals in GA, NC, and NY (Fig. 5). When SNPs on the four LD blocks are excluded from the analysis, individuals from GA, NC, and NY all show high assignment (approximately >75%) to population 1. Similarly, PCA of SNPs excluding those in LD blocks shows much less separation and more overlap of the three populations south of Cape Cod (Fig. S3). Furthermore, genome‐wide levels of differentiation south of Cape Cod appear to increase steadily with geographic distance, but the slope is far shallower when differentiation within LD blocks is excluded (Fig. S4). Cumulatively, these results indicate high connectivity across this region, with differentiation of the LD blocks accounting for most of the structure.

The 2dSFS, which summarizes the number of SNPs with a given frequency in two populations, can be shaped by both neutral and nonneutral forces. We compared the fit of 2dSFS generated from demographic models with and without gene flow, and including the heterogeneous effects of selection and differential introgression on the genome (Rougeaux et al. 2017), to the observed 2dSFS between GA and NY. All models that allowed for reduced effective population size (*N* *_e_*) across a proportion of the genome due to selection fit consistently better than models that did not, and models that included migration fit better than those that did not (Table S4; Fig. S5). The best ranking model was one of symmetrical migration with ∼12% of the genome having *N* *_e_* reduced to 0.013 that of the rest of the genome. Overall, the SFS of models that included the effects of selection were able to capture the highly differentiated alleles along the edges of the SFS (Figs. 5C and 5D), suggesting that these sites were under recurrent selection (Charlesworth 2009), whereas models that did not include heterogeneity in the genome underpredicted the frequency of these sites in the SFS (Fig. 5E).

The finding of ongoing migration between these two populations corroborates the more detailed demographic history reconstructed with Approximate Bayesian computation based on full mitochondrial genome sequences of these same individuals (Lou et al. 2018). Continuous migration models also outperformed secondary contact models, and the optimal estimate for the duration of time in isolation in the best models of secondary contact was small (Table S4). This structure differs from the classic clines in metabolic genes of *Fundulus heteroclitus* along the same coastline (Powers and Place 1978; Ropson et al. 1990), which occur across a geographic region shaped by secondary contact, making it difficult to fully disentangle effects of selection from demographic history in *F. heteroclitus* (Strand et al. 2012). In contrast, the fixation of opposite alleles among Atlantic silverside populations spans a coastline (GA to NY) with neither a historical mitochondrial lineage split (Lou et al. 2018) nor evidence of a period of divergence in isolation in the nuclear genome, and therefore is unlikely to be driven by secondary contact.

In addition to long‐term migration along the southern coastline, we observed four individuals with very rare haplotypes on outlier chromosomes relative to their capture location; at least two likely stem from recent migration events. One silverside from GSL (of *n* = 42) appears to have originated near NY based on transcriptome‐wide variation (Figs. 1A and S3B). It was homozygous for southern haplotypes on LD block 11 and heterozygous on LD block 24 (a combination of haplotypes observed in some NY individuals), and thus was likely a first‐generation migrant. In GA (*n* = 48), one individual was heterozygous on LD blocks 18 and 24, and comprised the only observation of northern haplotypes on these blocks in GA. Heterozygosity on two LD blocks suggests that this individual may be a recent cross between GA and NY (or an intermediate location). Two NY individuals (of *n* = 49) were heterozygous for only LD block 24 and no other blocks, indicating that they were either F2 backcrosses from a GA migrant or there is stable polymorphism of both LD block 24 haplotypes in NY. Estimates of intersource population ancestry of individuals from GA and NY support the inference that these heterozygous individuals are interpopulation hybrids resulting from recent admixture (i.e., have at least one nonadmixed parent; Fig. S2A), whereas the individual sampled in GSL shows high posterior assignment to NY with no evidence of interpopulation ancestry (Fig. S2B).

Observations of at least two recent long‐distance migration events out of fewer than 200 fish further support ongoing gene flow as an explanation for the limited neutral structure, and indicate not only local stepping‐stone migration, but also frequent long‐distance dispersal events that may stem from the winter migration offshore to the continental shelf at northern latitudes (Clarke et al. 2010). Evidence of historical connectivity through demographic modeling (Lou et al. 2018) as well as contemporary gene flow is bolstered by direct dispersal estimates based on otolith chemistry (Clarke et al. 2010). Elevated absolute divergence (*d_XY_*) within LD blocks suggests selection against maladaptive gene flow at these loci (Cruickshank and Hahn 2014). The nearly complete absence of alternate haplotypes across LD blocks, except in recent migrants or their offspring, between otherwise highly connected populations (e.g., GA and NY) indicates very rapid selection against migrant haplotypes at these loci.

### GREATER CLUSTERING OF OUTLIER LOCI ACROSS REGIONS WITH HIGH GENE FLOW

The geographic region most strongly connected by gene flow was where we found the most and the largest outlier blocks. Further north, gene flow appears to be more restricted, yet much smaller chromosomal regions show elevated differentiation. Prior phenotypic work demonstrates adaptive clines through both the northern and southern part of the Atlantic silverside range (Hice et al. 2012). Although the full genomic architecture underlying these adaptations remains to be uncovered, our findings here of more clustered genomic signatures of selection in the South are consistent with theoretical predictions that higher gene flow results in more linked architecture, usually by inversions (Kirkpatrick and Barton 2006). Support for this prediction has been shown at the interspecific level in *Drosophila*, passerines, rodents, and *Helianthus* sunflowers, where sympatric species with greater potential for introgression have more inversion polymorphisms that differentiate them than allopatric species (reviewed in Wellenreuther and Bernatchez 2018). The strongest evidence for intraspecific adaptive inversions comes from environmental clines, for example, inversions associated with climatic gradients in *Drosophila melanogaster*, *Anopheles* mosquitoes, and *Mimulus guttatus*, and with biotic and abiotic clines in *Littorina saxatilis* (Faria et al. 2018; Wellenreuther and Bernatchez 2018), reflecting inversions as a common mechanism for adaptive divergence along continuous environmental gradients.

### GENES IN LD BLOCKS HAVE FUNCTIONS RELATED TO MULTIPLE ADAPTATIONS

Near perfect LD across blocks of highly differentiated SNPs indicates that alleles at these loci are nearly always associated (even if not physically linked). To examine potential functions associated with the LD blocks, we tested for GO enrichment of genes within each of them relative to the rest of the genome. Highly differentiated genes (ranked by the number of OutFLANK outlier SNPs) in LD block 8 were enriched for genes involved in humoral immune response (26 significant terms in total; Table S5). Genes involved in renal filtration were also enriched in this block, possibly reflecting adaptation to local salinity conditions, as the kidney is a major osmoregulatory organ in teleosts (Marshall and Grosell 2006). LD block 18 was enriched for terms related to metabolic processes, mirroring variation in metabolic rates across latitudes in Atlantic silversides (Conover and Present 1990; Arnott et al. 2006), and a number of terms related to synapse maturation and neurotransmitter function, possibly reflecting temperature adaptations in nervous system function (Baldwin 1971; Montgomery and Macdonald 1990; Pörtner et al. 2007), as well as cartilage morphogenesis (29 significant terms; Table S6). On Chr. 24, enriched terms (of 47 significant terms) were related to regulation of lipid storage, reflecting lipid content variation across latitudes demonstrated by common garden experiments (Schultz and Conover 1997). Other notable enriched terms were related to development and differentiation of Sertoli cells, which play a central role in spermatogenesis in other fish (Rolland et al. 2009; Bahamonde et al. 2016), providing a potential link to variation in spawning periods in Atlantic silversides (Table S7; Conover and Kynard 1984). LD block 24 was also associated with body size in experimental silverside populations (Therkildsen et al. 2019), indicating a link to variation in growth rate. LD block 11 was enriched for genes that regulate T cell‐mediated immunity and other immune functions (22 significant terms; Table S8). Selective sweeps on immune genes have been shown in a number of other teleost species (e.g., Jensen et al. 2008; Kjærner‐Semb et al. 2016). Other notable terms enriched in LD block 11 overlapped with functions in other blocks, for example, glomerulus morphogenesis and lipid organization, and a number of significant GO terms in LD block 11 were related to cardiac function, known to be a limiting factor in temperature tolerance in other fish species (Steinhausen et al. 2008; Anttila et al. 2014), and may play a role in adaptation to the steep thermal cline along the Atlantic coast.

These functional enrichment analyses suggest that the highly differentiated LD blocks may underlie some of the trait divergence already described for the Atlantic silverside. Prior work on both wild and experimental silverside populations have suggested strong correlations among locally adapted traits (Walsh et al. 2006; Hice et al. 2012; Salinas et al. 2012). Artificial selection on growth rate resulted in concomitant shifts in other traits related to growth efficiency, morphology, and fecundity (Walsh et al. 2006; Salinas et al. 2012). Our results here provide an indication that genes underlying multiple complex traits may co‐locate to the same LD blocks, potentially representing co‐adapted gene complexes (Kirkpatrick and Barton 2006).

It is important to point out that if these LD blocks are associated with inversions, genes within the inversions are not independent. It is possible that clusters of genes with similar functions (from tandem duplications, e.g.) captured within an inversion may lead to apparent enrichment of those functions simply because the genes are physically linked. Additional research is underway to determine whether the LD blocks are associated with chromosomal inversions, as well as genotype‐phenotype‐environment analyses with increased sampling along the latitudinal cline to tease apart the targets of selection. Nonetheless, multiple lines of evidence point to an adaptive role for the LD blocks, and the functional enrichment results give an indication of the major functions of genes within them, even if their adaptive roles remain unclear.

### IMPLICATIONS

Virtually all the traits known to vary genetically in Atlantic silversides are now known to do so in numerous other species (Conover et al. 2005). The genomic patterns generated by local adaptation in silversides are an extreme case of what may be a common feature of wide‐ranging populations distributed across highly connected environmental gradients; thus, Atlantic silversides are a useful model not only for studying adaptive variation vital to preserving population health and adaptive potential, but also for identifying and conserving functional variation in managed and harvested marine species. Although the Atlantic silverside is only caught in commercial fisheries to a limited extent, this species demonstrates how even the most striking adaptations may be cryptic: field observations of similar sizes at maturity across the range mask huge adaptive variation in this key life history trait (Conover and Schultz 1995), and even the megabase‐scale haplotypes underlying local adaptation were not detected when marker density was low (Mach et al. 2011). Without information from laboratory experiments and genomic analysis, low genome‐wide differentiation may prompt a decision to manage highly divergent alleles and phenotypes as if they were a nearly panmictic unit, when managing them as interdependent populations with unique adaptive variation would be warranted. This example underscores the importance of incorporating large‐scale genomic data (and, when possible, common garden experiments) to identify and manage adaptive variation, particularly in widely distributed species.

Our findings also highlight the critical interplay between gene flow and selection in shaping the genomic architecture that lays the foundation for the response to future environmental shifts. Artificial selection has demonstrated the high adaptive potential of Atlantic silversides (Conover and Van Voorhees 1990; Conover and Munch 2002), and shown that the genomic variation leveraged for rapid adaptation depends on standing variation available in the population (Therkildsen et al. 2019). Gene flow may accelerate an adaptive response to climate change by providing relevant genetic variation, especially along a thermal gradient. However, tight linkage of co‐adapted alleles, an architecture associated with selection under gene flow (Kirkpatrick 2010; Samuk et al. 2017), may also constrain an adaptive response if linked traits are antagonistic to the direction of selection (Etterson and Shaw 2001). Beneficial alleles linked to maladaptive traits will not rapidly dissociate if recombination is suppressed between alternate alleles. This interplay is becoming increasingly relevant as humans alter both the environment and natural levels of gene flow, and organisms must rapidly adapt in response.

## AUTHOR CONTRIBUTIONS

NOT and SRP designed the study. NOT and APW generated the data. APW and NOT analyzed the data with input from SRP. DOC collected the samples. All authors wrote the manuscript.

## DATA ARCHIVING

The genomic sequence data described in this article have been archived and are publicly available in the NCBI Short Read Archive under Bioproject ID PRJNA376564. Sample locality data and transcriptome‐wide SNP data can be accessed through the Dryad repository https://doi.org/10.5061/dryad.jm63xsj7w.

## Supporting information


**Table S1**. Nucleotide diversity statistics within populations of Atlantic silversides based on pairwise differences (π) and number of polymorphic sites (Watterson's θ) across the transcriptome.
**Table S2**. Pearson's χ2 contingency tables and goodness of fit significance tests for enrichment of variant types in the top 1% of the FST distribution between pairs of neighboring populations relative to the genome‐wide distribution of variant types.
**Table S3**. Pearson's χ2 contingency tables and goodness of fit significance tests for enrichment of untranslated region (UTR), non‐synonymous (NS), or synonymous (S) variant types in LD blocks 8, 18, 24 and 11 (D‐G), relative to the genome‐wide distribution of variant types.
**Table S4**. Fits and parameter estimates for demographic models of the 2dSFS of GA and NY, showing the best of five independent runs ranked by AIC.
**Table S5**. Enrichment of Biological Processes Gene Ontology (GO) terms among genes scored by summing the number of OutFLANK outlier SNPs in LD block 8.
**Table S6**. Enrichment of Biological Processes Gene Ontology (GO) terms among genes scored by summing the number of OutFLANK outlier SNPs in LD block 18.
Table S7. Enrichment of Biological Processes Gene Ontology (GO) terms among genes scored by summing the number of OutFLANK outlier SNPs in LD block 24.
**Table S8**. Enrichment of Biological Processes Gene Ontology (GO) terms among genes scored by summing the number of OutFLANK outlier SNPs in LD block 11.
**Figure S1**. Pairwise linkage disequilibrium (LD) between outlier SNPs on silverside contigs (genes) in A) North Carolina (NC), B) New York (NY), and C) Gulf of Maine (GoM).
**Figure S2**. A) Interpopulation ancestry analysis of individuals from NY and GA showing high interpopulation ancestry of one individual from GA (the individual that was heterozygous for southern and northern alleles on Chr 8, 18, and 24), and two NY individuals (heterozygous for southern and northern alleles on Chr 24).
**Figure S3**. Principal components (PC) 1 and 2 using A) all SNPs, B) all SNPs outside of LD blocks, and C‐F) SNPs in LD blocks 8, 18, 24, and 11. Note that for Blocks 18, 24, and 11, individuals cluster into three distinct groups on PC1, likely corresponding to the two homozygous and the heterozygous state for each LD block.
**Figure S4**. Isolation by distance between all pairs of silverside populations, showing differentiation across genome‐wide SNPs (circles) and differentiation of SNPs outside LD blocks (triangles).
**Figure S5**. Fit of demographic models to the joint SFS of silversides from GA and NY, colored by the number of SNPs in each combination of minor alleles in GA and NY.
**Figure S6**. Fit of the top‐ranked demographic model (symmetrical migration with heterogeneous Ne) to the empirical 2dSFS for GA and NY (blue vertical line), relative to 100 Poisson‐sampled 2dSFS simulated under the model (gray bars), showing a good fit of the model to the data.
