# Supplementary material for: Footprints of local adaptation span hundreds of linked genes in the Atlantic silverside genome
Source: Evol Lett. 2020 Aug 19;4(5):430–43. doi: 10.1002/evl3.189 (PMC7523562; doi:10.1002/evl3.189)
Supplement: Supplementary file 1 — Table S1. Nucleotide diversity statistics within populations of Atlantic silversides based on pairwise differences (π) and number of polymorphic sites (Watterson's θ) across the transcriptome. Table S2. Pearson's χ2 contingency tables and goodness of fit significance tests for enrichment of variant types in the top 1% of the FST distribution between pairs of neighboring populations relative to the genome‐wide distribution of variant types. Table S3. Pearson's χ2 contingency tables and goodness of fit significance tests for enrichment of untranslated region (UTR), non‐synonymous (NS), or synonymous (S) variant types in LD blocks 8, 18, 24 and 11 (D‐G), relative to the genome‐wide distribution of variant types. Table S4. Fits and parameter estimates for demographic models of the 2dSFS of GA and NY, showing the best of five independent runs ranked by AIC. Table S5. Enrichment of Biological Processes Gene Ontology (GO) terms among genes scored by summing the number of OutFLANK outlier SNPs in LD block 8. Table S6. Enrichment of Biological Processes Gene Ontology (GO) terms among genes scored by summing the number of OutFLANK outlier SNPs in LD block 18. Table S7. Enrichment of Biological Processes Gene Ontology (GO) terms among genes scored by summing the number of OutFLANK outlier SNPs in LD block 24. Table S8. Enrichment of Biological Processes Gene Ontology (GO) terms among genes scored by summing the number of OutFLANK outlier SNPs in LD block 11. Figure S1. Pairwise linkage disequilibrium (LD) between outlier SNPs on silverside contigs (genes) in A) North Carolina (NC), B) New York (NY), and C) Gulf of Maine (GoM). Figure S2. A) Interpopulation ancestry analysis of individuals from NY and GA showing high interpopulation ancestry of one individual from GA (the individual that was heterozygous for southern and northern alleles on Chr 8, 18, and 24), and two NY individuals (heterozygous for southern and northern alleles on Chr 24). Figure S3. Principal components (PC) [file EVL3-4-430-s001.docx]

**Supplemental Material**

Footprints of local adaptation span hundreds of linked genes in the Atlantic silverside genome

Wilder, Aryn P.; Palumbi, Stephen R.; Conover, David O.; Therkildsen, Nina O.

Supplemental Methods and Results:

*Read mapping and SNP calling*

For samples from GA, NY, GoM and GSL that all were sequenced by Therkildsen & Palumbi (2017), read adapters were trimmed from the paired-end 125 bp reads using Trimmomatic v.0.3.6 (Bolger et al. 2014), and mapped to the reference transcriptome with Bowtie2 v2.2.3 (Langmead and Salzberg 2012), using the very-sensitive-local mode and retaining unpaired, orphaned and concordantly paired reads with mapping quality >20. Duplicate reads were removed with MarkDuplicates (PICARD Tools v1.139; http://broadinstitute.github.io/picard/). See Therkildsen & Palumbi (2017) and Therkildsen et al. (2019) for further detail. Mapped reads had a final, average depth of 1.5x across the reference transcriptome.

We added 47 additional samples from Oregon Inlet, North Carolina (NC) near Cape Hatteras. In Therkildsen et al. (2019), we found a block of SNPs that mapped to medaka Chr 24 to be fixed for either the reference or alternate allele in all our four sampling locations, thus we added the NC location located between GA and NY, where we expected allele frequencies to be intermediate, hence allowing analysis of linkage disequilibrium (LD). Individually barcoded sample libraries were prepared at Cornell’s Biotechnology Resource Center using similar methods to Therkildsen & Palumbi (2017), with a few minor modifications. Reagents from the Illumina Nextera kit (96 sample Nextera DNA Library Prep Kit) were used at 1/3 the recommended concentration in 1/10 the recommended volume (5ul instead of 50ul), with 2ng of input DNA. Individual libraries were pooled, and size-selected using a Pippin Prep to remove fragments <286 bp (150 bp insert plus 136 bp of Illumina adapters). We generated paired-end 75 bp sequences on the NextSeq500. Data cleaning and mapping was performed as described above, and filtered mapped reads for the NC samples had a final average depth of 1.5x.

To avoid spurious SNP calls stemming from differences in read length in the NC sample (Leigh et al. 2018), we called SNPs across the original four population sample set (GA, NY, GoM and GSL). Downstream analyses of all samples (including NC) were performed on this set of SNPs. Because NC is located in the center of the distribution of the southern phylogeographic group (Mach et al. 2005; Lou et al. 2018), we do not expect to have missed variants private to that population by excluding it from the SNP calling. Biallelic SNPs were called in the program ANGSD v. 0.912 (Korneliussen et al. 2014). ANGSD estimates genotype likelihoods at each site based on the aligned reads and their mapping and sequencing quality scores. Using genotype likelihoods for low-coverage sequencing data allows for the incorporation of uncertainty in genotyping, providing more accurate population genetic inference (Li 2011; Nielsen et al. 2011). We called polymorphic sites across the four original populations (*n* = 189), at sites with a probability <1e-6 of being monomorphic, using the SAMtools model of genotype likelihoods in ANGSD. Bases with quality score <20 were excluded. We excluded sites with minor allele frequency (MAF) < 0.01, total read depth <75 and >759 (mean depth +1 standard deviation), or data from <75 individuals. ANGSD called 1,942,329 SNPs that passed the above filters. From these, we filtered out 38,210 SNPs within repetitive elements identified by RepeatMasker (Smit et al. 2017). The final dataset comprised 1,904,119 bialleleic SNPs across the ~52 MB transcriptome. Contigs had a mean of 92.57 SNPs, and an average of 0.04 SNPs per bp (1 SNP per ~24 bp).

*Ordering Atlantic silverside* *genes along medaka chromosomes*

We downloaded all medaka peptide sequences (Ensembl release 75; Cunningham et al. 2015) and used blastx to compare silversides contigs to medaka peptides . We then compared medaka peptides to silverside contigs using tblastn with soft masking and an e-value < 10^-4^ (Altschul et al. 1990, 1997). 19,230 of the 20,998 contigs had a reciprocal best hit to the medaka genome. Of these, 17,724 contigs mapped to one of the 24 medaka chromosomes. Chromosomes had a mean of 738.5 mapped contigs (range = 339 – 959), and a mean of 86,156 SNPs (range = 39,002 – 114,992).

*Functional annotation*

Contigs in the reference transcriptome were annotated with the Blast2Go v3.1.2 suite (Conesa et al. 2005). For each sequence, we imported significant hits (e-value < 1e-6) from blastx searches against the UniProt Swiss-Prot and the NCBI non-redundant (NR) protein databases. We used Blast2Go’s Blast Description Annotator tool to select the most informative and relevant descriptor before mapping Gene Ontology (GO) terms to the matches and applying the built-in annotation rules with default parameters. We also imported GO-terms associated with reciprocal-best-hit genes in three reference fish species (Xiphophorus maculatus, *Oryzias latipes*, *Oreochromis niloticus*) and merged the combined sets of assigned annotations with GO-terms inferred from InterProScan analysis of each sequence (Jones et al. 2014). As a final step, we used the Blast2Go Validate Annotations tool to ensure that no parent-child redundancy was present in the assigned GO-terms, and we applied the Annex tool to augment the annotation based on inference of biological processes from commonly associated molecular functions and cellular components (Myhre et al. 2006).

To understand the potential function of SNPs within the transcriptome, we used two programs, Transdecoder and GeneMarkS-T, to predict coding sequences (CDS) and untranslated regions (UTR). From these annotations, we used snpEff to predict the function (e.g., missense variant, synonymous variant, 3’ UTR variant) of each SNP. Variant types predicted by SNPeff were classified as UTR (3 prime UTR variant, 5 prime UTR variant, downstream gene variant, downstream gene variant, intergenic region, splice region variant, 3 prime UTR variant, upstream gene variant, upstream gene variant, intergenic region, 5 prime UTR premature start codon gain variant, 5 prime UTR variant), non-synonymous (missense variant, missense variant & splice region variant, start lost, stop gained, stop lost, stop lost & splice region variant), or synonymous (initiator codon variant, initiator codon variant & non-canonical start codon, splice region variant & stop retained variant, splice region variant & synonymous variant, stop retained variant, synonymous variant, 5 prime UTR premature start codon gain variant).

*SNP and annotation quality control*

Data from both SNP variant types and the inferred location of intron-exon boundaries were used to verify our CDS predictions and confirm that mapping genomic reads to the transcriptome (instead of a reference genome sequence) did not result in spurious SNP calls. The reference transcriptome lacks intronic regions, and thus genomic reads that spanned an intron-exon boundary (IEB) contained both sequence from the exon (which maps to the reference transcriptome), and sequence from the intron (which was soft-clipped during read mapping). In most cases the position of IEBs could be readily identified by the large number of read alignments that start at a given position (see Therkildsen et al 2019 supplementary materials).

There were 209,317 missense variants (27.2% of CDS variants) and 557,553 synonymous variants (72.7% of CDS variants) in total, and the median proportion of missense variants across all CDS was similar to the mean (22.2% versus 26.3%), indicating that the distribution of missense variants is not skewed by bad CDS predictions that yield many missense variant predictions. To assess the impact of alignment errors near IEBs resulting from mapping genomic reads to the transcriptome, we evaluated the number of missense variants within 5 bases of each IEB. We found that 5.5% of all missense variants occurred within 5 bp of an IEB. In turn, IEB windows comprised ~5.1% of the transcriptome; thus missense variants were proportionally only slightly higher adjacent to IEBs relative to the rest of the transcriptome, and account for a small proportion of the total variants.

*Estimating allele frequencies and identifying F_ST_ outliers*

At all globally variant sites, major and minor alleles were inferred based on global allele frequencies and minor allele frequencies (MAF) were estimated within populations from genotype likelihoods at sites with data for at least 10 individuals in the program ANGSD. We estimated pairwise F_ST_ at each SNP between population pairs using the 2-dimensional site frequency spectrum (2D SFS) as prior. To estimate the number of F_ST_ outlier SNPs in the transcriptome, we compared observed global F_ST_ values from allele frequencies at all variant sites to an expected χ^2^ distribution fit to a subset of randomly sampled SNPs. The subset was obtained by pruning a set of 50,000 randomly sampled SNPs for linkage disequilibrium (LD) across the global population using default settings in the program ngsLD (Fox et al. 2019). We removed SNPs with weight *D*>0.1 using the SNP pruning function of ngsLD, and also removed SNPs with heterozygosity < 0.2, yielding 32,385 SNPs. For each SNP, we estimated the global F_ST_ across all five populations as in the OutFLANK script *FST functions.R*, except that we made a slight modification to the script so that we could directly input allele frequencies and sample sizes estimated in ANGSD (which takes genotype likelihoods into account), rather than estimating allele frequencies from allele counts in OutFLANK. F_ST_ was then estimated by the procedure implemented in OutFLANK. We fit the χ^2^ distribution to the pruned SNP set by trimming 5% from each side of the F_ST_ distribution and using *q*<0.05 to estimate the degrees of freedom and F_ST_bar of the χ^2^ distribution in OutFLANK (Whitlock and Lotterhos 2015). We then applied the χ^2^ fit to all SNPs across the transcriptome to identify F_ST_ outliers using *q*<0.05.

*Identifying LD blocks*

We estimated linkage disequilibrium between all pairs of SNPs identified as outliers in the OutFLANK analysis. Because the majority of these SNPs were fixed for opposite alleles at the extremes of the geographic distribution, precluding LD inference, we limited our LD analysis to the three sampling locations in the center of the range (NC, NY and GoM). Within each of these populations, we estimated LD across pairs of SNPs with MAF>0.1 in the program ngsLD (Fox et al. 2019). Because the result consisted of more than 200 million pairwise LD estimates between SNPs (and we know that SNPs on the same contig are physically linked), we summarized patterns by calculating an average LD between pairs of contigs that had at least 2 outlier SNPs with MAF>0.1. For each pair of these contigs, we calculated the mean D’ across all pairs of outlier SNPs between contigs. We used a hierarchical clustering approach to determine how contigs clustered into blocks within each population by generating dendrograms from the pairwise LD matrix using the “Ward.D” method in the hclust function in R (Murtagh and Legendre 2014). In NC, the dendrogram created from all contigs polymorphic in that population (at least 2 outliers SNPs with MAF>0.1) shows four clear branches (Figure S1A). Three of the four branches form tight LD clusters, and the majority of genes in each of these clusters map primarily to a single unique medaka chromosome. The largest cluster of 391 contigs had a mean D’=0.89, and 91.2% of contigs that mapped to a medaka chromosome mapped to Chr 24. A second cluster of 255 contigs had mean D’=0.86, and 82.3% of mappable contigs in this cluster mapped to Chr 18. A third cluster of 263 contigs had mean D’=0.79, and 89.5% of mappable contigs corresponded to medaka Chr 8. A fourth branch grouped 435 contigs with relatively low LD (mean D’=0.68), and no medaka chromosome was clearly represented in the group (Figure S1A). In GoM, the dendrogram formed two branches, one of which clustered a block of 263 genes with high LD (mean D’=0.82), whereas the other branch was a cluster of relatively unlinked contigs (mean D’=0.61; Figure S1C). Genes in the high LD cluster largely mapped to medaka Chr 11 (72.9% of mappable contigs). Similarly, LD across contigs in NY formed two clear branches; however, many contigs were excluded because of low polymorphism at these contigs in this population. Nonetheless, 184 out of 187 contigs in the high LD block were also present in the LD cluster in GoM corresponding to medaka Chr 11. For all subsequent analyses, we defined four LD blocks by the membership of contigs to the three high LD branches in NC, and the one high LD branch in GoM. Of 1,710 contigs in the hierarchical clustering analysis, 1,171 assigned uniquely to one of these LD blocks (hereafter referred to as LD blocks 8, 11, 18 and 24), and 539 contigs were not part of an LD block. The membership of genes in LD blocks was defined exclusively based on LD patterns within single populations without any a priori information about gene locations in the medaka genome, and thus the high degree of consistency between LD block membership and chromosomal position within the medaka genome suggest that the contigs within each block are very likely physically linked on separate chromosomes in the silverside genome.

In order to visualize individual genotype patterns across LD blocks, we generated heatmaps showing the most likely genotype for each fish based on genotype likelihoods at outlier SNPs in each LD block. Genotype likelihoods were estimated for each of the three possible genotypes (the heterozygous and two homozygous genotypes), and normalized to sum to one (beagle formatted output in ANGSD). For each SNP, we defined the “southern” allele as the major allele in GA, and multiplied the homozygous southern genotype likelihood by two, the heterozygous genotype likelihood by one, and then summed the two values to estimate the number of southern alleles at each position for each individual. The number of southern haplotypes carried by each individual were estimated by the average number of southern alleles across all SNPs (left barplots in Figure 4). To simplify visualization of genotype patterns in heatmaps, genotypes (i.e. number of southern alleles) at each site were rounded to the nearest integer for plotting.

*Population genomic analyses*

Watterson’s theta and pairwise nucleotide diversity were estimated from the folded SFS (which does not require knowledge of the ancestral state of each variant). To account for differences in the number of samples across populations, we randomly subset the number of individuals in each population to 41. We then estimated the folded SFS for each population in the program ANGSD using parameter settings described above for allele frequency estimation, except that we applied no MAF filter or p-value threshold for polymorphism. Thetas were estimated for each site using the doThetas function in Angsd, passing the folded SFS as a prior. The per-site thetas were averaged to obtain the genome-wide estimates.

Admixture analysis was performed using default settings in the program PCAngsd (Meisner and Albrechtsen 2018), which uses genotype likelihood data to find the value of K that best fits the data, then estimates posterior assignment probabilities to each cluster. We ran the admixture analysis on all 1,904,119 biallelic SNPs across all individuals, then reran the analysis on all SNPs, excluding contigs in the four LD blocks. For both sets of SNPs, the optimal model was K=3. When all SNPs were included, there was a gradient in posterior assignment to populations 1 and 2 of individuals in GA, NC and NY (Figure 5), but when SNPs on the four LD blocks are excluded from the analysis, individuals from GA, NC and NY all show high assignment (>~75%) to population 1 (Figure 5).

To detect interpopulation hybrids, we estimated interpopulation ancestry and average ancestry in the program *entropy* (Gompert et al. 2014). *Entropy* implements an admixture class model using genotype likelihoods to distinguish contemporary versus historical admixture. We used a random subset of 100,000 SNPs under K=4 (we used K=4 and excluded the NC population so that we could better distinguish GA from NY) to determine the interpopulation hybrid status of potential migrant individuals from GA, NY and GSL identified by locally rare haplotypes in the LD blocks and by the admixture analysis. The GA individual with the highest estimate of interpopulation ancestry (indicative of a recent, non-admixed parent; Figure S2A) was heterozygous for northern and southern haplotypes in LD blocks 8, 18 and 24, and two NY individuals with non-zero interpopulation ancestry were heterozygous for LD block 24 (Figure S2A; Figure 3). By contrast, the GSL individual with high posterior assignment to NY showed no evidence of interpopulation ancestry (Figure S2B).

Principal components analysis (PCA) was performed by computing eigenvectors in R from the covariance matrix between individuals estimated from a given set of SNPs in PCAngsd (Meisner and Albrechtsen 2018). To explore different patterns of structure across the genome, we ran PCAs on 1) all transcriptome-wide SNPs, 2) all SNPs excluding the LD blocks, and 3) the SNPs in each LD block separately. Whereas PC plots of all SNPs shows a cline of differentiation between GA, NC and NY (Figure 1; Figure S3A), PCs of SNPs outside of LD blocks show much overlap of samples from GA and NC, and little separation between NC and NY (Figure S3B). PCAs using SNPs in LD blocks 18, 24 and 11 had high proportions of variation captured by PC1 (92.5%, 86.0% and 54.7%, respectively), and samples tended to fall into three groups along PC1 corresponding to homozygous southern, heterozygous, and homozygous northern genotypes (Figure S3D-F). For LD block 8 (Figure S3C), which had generally lower LD than the other LD blocks, PC1 captured 25.1% of the variation, and samples fell into a larger number of groups along PC1.

We evaluated the relationship between differentiation and geographic distance between populations for all transcriptome-wide SNPs (including those in LD blocks) and for all SNPs outside LD blocks (Figure S4). Geographic distance was estimated as least-cost geographic distances between the silverside sampling locations, taking landmass and depth profiles into account. Genetic distance was estimated as the mean F_ST_ across SNPs, shown as F_ST_/(1-F_ST_). Genome-wide levels of differentiation south of Cape Cod appear to increase steadily with geographic distance, but the slope is far shallower when differentiation within LD blocks is excluded (Figure S4).

*Demographic modeling*

Despite having three megabase-scale LD blocks nearly fixed for opposite alleles, GA and NY had the lowest genome-wide F_ST_ and previous demographic modeling based on full mitochondrial genome sequences suggested that the two populations remained connected through the last glacial maximum (Lou et al. 2018). In order to test the hypothesis that there is gene flow between populations (even those with divergent haplotypes across extended LD blocks), we compared the fit of simple demographic models to the observed 2-dimensional site frequency spectrum (2dSFS) for GA and NY. We first estimated allele frequencies within these two populations at all sites using the same parameter settings in ANGSD described above, except that we applied no MAF filter or p-value threshold for polymorphism, and we applied more stringent filters on the minimum number of individuals and reads (minInd = 22 and minDP = 48 in each population). In each population, the number of alleles was estimated at all sites by multiplying the frequencies of the reference and alternate alleles by the number of individuals for which there was data at the site (both output by ANGSD). To minimize effects of linkage across SNPs, we randomly sampled one SNP from each contig in the transcriptome to generate the 2dSFS (16,189 SNPs in total).

We used the moments_pipeline to fit demographic models to the 2dSFS (Leaché et al. 2019). The pipeline implements the program moments (Jouganous et al. 2017), which uses differential equations to simulate the evolution of allele frequency distributions over time and is closely related to the diffusion approximation method used in the program ∂a∂i (Gutenkunst et al. 2009). We compared the fit of the observed 2dSFS to those simulated under eight evolutionary models for the two populations (Table S4). Each model consisted of an ancestral population of size Na that split into two populations of effective size N1 and N2 at time T in the past, with the following migration scenarios: 1) no migration; 2) symmetrical migration; and 3) secondary contact (in which the populations diverge in isolation for duration of time T1 at time T1+T2 in the past, followed by symmetrical migration m starting at T2 in the past). We extended each of these three basic scenarios in models allowing for heterogeneity in Ne across the genome due to the effect of background/positive selection, and extended the two basic models with gene flow to allow for heterogeneous migration across the genome (Rougeux et al. 2017). For the three models including heterogeneous Ne, a second category of loci encompassing Q proportion of the genome have Ne reduced by hrf, a Hill–Robertson scaling factor relating the effective population size of loci influenced by selection (N’1=hrf x N1 and N’2=hrf x N2) to that of neutral loci (N1 and N2; Rougeux et al. 2017). For the two models of divergence with gene flow, heterogeneous migration across the genome was modeled by estimating an additional migration parameter (m’) for a second category of loci encompassing P proportion of the genome.

We imported the allele counts (generated from ANGSD output) and estimated the folded 2dSFS in the moments_pipeline, projecting the data down to 54 and 56 alleles for GA and NY, respectively. We chose the projection that maximized the number of segregating sites in the 2dSFS (Gutenkunst et al. 2009). The 2dSFS after projection had 13769.6 sites. For all models, we performed five independent runs of consecutive rounds of optimizations with multiple replicates within each round. For each independent run, parameter estimates from the best scoring replicate (highest log-likelihood) were used to seed searches in the following round. We used the default settings for optimization in the moments_pipeline for each round (replicates = 10, 20, 30, 40; maxiter = 3, 5, 10, 15; fold = 3, 2, 2, 1). We set the lower bound to 0.01 for population size parameters, and to 0 for all other parameters. We set the upper bound for population size parameters to 20, migration parameters to 50, time parameters to 10, and proportional parameters (hrf, P and Q) to 1. The optimized parameter sets of each replicate were used to simulate the 2dSFS, and the multinomial approach implemented in moments was used to estimate the log-likelihood of the 2dSFS given the model. We ranked models by AIC, and tested the goodness of fit of the top model using the Simulate_and_Optimize.py scripts in the moments_pipeline, simulating 100 Poisson-sampled 2dSFS under the model using the optimized parameter values (Leaché et al. 2019).

Of the eight models tested, all three models that allowed for reduced Ne across a proportion of the genome due to selection fit consistently better than models that did not, and models that included migration fit better than those that did not (Table S4; Figure S5). The best ranking model by AIC was one of symmetrical migration with ~12% of the genome having Ne reduced to 0.013 that of the rest of the genome, suggesting the effects of selection to locally reduce diversity at affected loci. The fit of the model to the empirical data was similar to that of 100 2dSFS simulated under the model (Figure S6), suggesting a good fit of the model to the data. To infer the scale of these parameter estimates, we applied a mutation rate of mu=5e-8 (Künstner et al. 2016), and considering that our 2dSFS had 13,770 SNPs of the ~1.9M SNPs discovered across the ~48M bp of the transcriptome used for SNP discovery, we applied an effective sequence length of 347,863 bp to estimate an ancestral population size of Na = 17,227. From Na, we scaled the parameter estimates into biologically meaningful units: Ne = 92,377 and 100,193 for GA and NY, respectively, T = 7,616 generations in the past, m = 7.17e-05 (or 6-7 effective migrants per generation in each population). For ~12% of the genome, Ne is reduced to 0.013 of the genome-wide Ne, and the effective number of migrants per generation is effectively 0 individuals (~0.09) in each population. Although we report the parameter estimates converted into biologically meaningful units here, we caution that the silverside specific mutation rate is unavailable. Our estimates of effective population size differ by an order of magnitude from Lou et al. (2018), which may reflect differences in mutation rates in the mitochondrial genome compared to the transcriptome, or differences in modeling methods.

*Functional enrichment*

We tested for enrichment of gene ontology (GO) terms among genes in each LD block. Each gene was assigned a score by summing the number of SNPs identified as F_ST_ outliers in OutFLANK. We also tested for enrichment of GO terms in the most highly differentiated genes between pairs of populations by scoring each gene with the sum of SNPs in the top 1% of genome-wide F_ST_. Because there was very little relationship between the number of high F_ST_ SNPs and the number of SNPs overall in each contig (*r*^2^ = 0.021), we did not correct the counts for the size of the contig. Enrichment of GO terms was tested using gene score resampling (GSR) analysis in the program *ErmineJ* (Gillis et al. 2010). We limited the GO terms evaluated by *ErmineJ* to those annotated with 10-900 genes (8,554 Biological Processes terms), and generated *p*-values from 200,000 iterations to correct for multiple testing.

We tested for enrichment of non-synonymous (NS) and untranslated region (UTR) variants compared to synonymous variants in the top 1% tail of the F_ST_ distribution, and among SNPs in LD blocks, using χ^2^ goodness-of-fit tests relative to the proportion of these variant types across the transcriptome.

Supplemental Tables:

| Table S1. Nucleotide diversity statistics within populations of Atlantic silversides based on pairwise differences (𝜋) and number of polymorphic sites (Watterson’s 𝛳) across the transcriptome. Note that these diversity estimates are based on transcriptome regions only. Levels of diversity across the entire genome (including intergenic regions) are likely to be higher. | | | | | |
| --- | --- | --- | --- | --- | --- |
|  | GA | NC | NY | GoM | GSL |
| 𝛳 | 8.82E-3 | 9.04E-3 | 8.88E-3 | 8.23E-3 | 7.51E-3 |
| 𝜋 | 1.03E-2 | 1.55E-2 | 1.01E-2 | 6.53E-3 | 5.66E-3 |

| Table S2. Pearson's χ^2^ contingency tables and goodness of fit significance tests for enrichment of variant types in the top 1% of the *F*_ST_ distribution between pairs of neighboring populations relative to the genome-wide distribution of variant types. | | |
| --- | --- | --- |
| 1. GA vs. NY | | |
| χ^2^ = 251, df = 1, p < 2.2e-16 | | |
|  | NS | S |
| Expected | 2141.01 | 5631.99 |
| Observed | 2765 | 5008 |
| % Enrichment | 29.14 | -11.08 |
|  |  |  |
| χ^2^ = 326.32, df = 1, p < 2.2e-16 | | |
|  | UTR | S |
| Expected | 9628.42 | 6112.58 |
| Observed | 10733 | 5008 |
| % Enrichment | 11.47 | -18.07 |
|  |  |  |
| 1. GA vs. NC | |  |
| χ^2^ = 236.03, df = 1, p < 2.2e-16 | | |
|  | NS | S |
| Expected | 2108.51 | 5546.49 |
| Observed | 2709 | 4.95E+03 |
| % Enrichment | 28.48 | -10.83 |
|  |  |  |
| χ^2^ = 321.63, df = 1, p < 2.2e-16 | | |
|  | UTR | S |
| Expected | 9507.31 | 6035.69 |
| Observed | 10597 | 4.95E+03 |
| % Enrichment | 11.46 | -18.05 |
|  |  |  |

| Table S2 (continued) | | |
| --- | --- | --- |
| 1. NC vs. NY | | |
| χ^2^ = 107.27, df = 1, p < 2.2e-16 | | |
|  | NS | S |
| Expected | 2497.43 | 6569.57 |
| Observed | 2938 | 6129 |
| % Enrichment | 17.64 | -6.71 |
|  |  |  |
| χ^2^ = 6.579, df = 1, p = 0.010 | | |
|  | UTR | S |
| Expected | 9410.05 | 5973.95 |
| Observed | 9255 | 6129 |
| % Enrichment | -1.65 | 2.60 |
|  |  |  |
| 1. NY vs. GoM | | |
| χ^2^ = 2.1138, df = 1, p = 0.146 | | |
|  | NS | S |
| Expected | 2226.39 | 5856.61 |
| Observed | 2168 | 5915 |
| % Enrichment | -2.62 | 1.00 |
|  |  |  |
| χ^2^ = 45.868, df = 1, p = 1.265e-11 | | |
|  | UTR | S |
| Expected | 9981.36 | 6336.64 |
| Observed | 10403 | 5915 |
| % Enrichment | 4.22 | -6.65 |
|  |  |  |
| 1. GoM vs. GSL | | |
| χ^2^ = 57.956, df = 1, p = 2.681e-14 | | |
|  | NS | S |
| Expected | 2293.33 | 6032.67 |
| Observed | 1983 | 6343 |
| % Enrichment | -13.53 | 5.14 |
|  |  |  |
| χ^2^ = 3.704, df = 1, p = 0.054 | | |
|  | UTR | S |
| Expected | 10181.99 | 6464.01 |
| Observed | 10303 | 6343 |
| % Enrichment | 1.19 | -1.87 |

| Table S3. Pearson's χ^2^ contingency tables and goodness of fit significance tests for enrichment of untranslated region (UTR), non-synonymous (NS), or synonymous (S) variant types in LD blocks 8, 18, 24 and 11 (D-G), relative to the genome-wide distribution of variant types. | | | | |
| --- | --- | --- | --- | --- |
| **A) LD block 8** | | | | |
| χ^2^ = 359.21, df = 1, p < 2.2e-16 | | | | |
|  | UTR | | S | |
| Expected | 17321.58 | | 11207.42 | |
| Observed | 18885 | | 9644 | |
| % enrichment | 9.03 | | -13.95 | |
|  | | | | |
| χ^2^ = 0.40, df = 1, p = 0.53 | | | | |
|  | NS | | S | |
| Expected | 3636.49 | | 9676.51 | |
| Observed | 3669 | | 9644 | |
| % enrichment | 0.89 | | -0.34 | |
| **B) LD block 18** | | | | |
| χ^2^ = 753.06, df = 1, p < 2.2e-16 | | | | |
|  | **UTR** | | **S** | |
| Expected | 13127.34 | | 8493.66 | |
| Observed | 15098 | | 6523 | |
| % Enrichment | 15.01 | | -23.20 | |
|  | | | | |
| χ^2^ = 477.30, df = 1, p < 2.2e-16 | | | | |
|  | **NS** | | **S** | |
| Expected | 2823.31 | | 7512.69 | |
| Observed | 3813 | | 6523 | |
| % Enrichment | 35.05 | | -13.17 | |
| **C) LD block 24** | | | | |
| χ^2^ = 459.64, df = 1, p < 2.2e-16 | | | | |
|  | **UTR** | | **S** | |
| Expected | 16339.20 | | 10571.80 | |
| Observed | 18083 | | 8828 | |
| % Enrichment | 10.67 | | -16.49 | |
|  | | | | |
| χ^2^ = 130.08, df = 1, p < 2.2e-16 | | | | |
|  | **NS** | | **S** | |
| Expected | 3534.88 | | 9406.12 | |
| Observed | 4113 | | 8828 | |
| % Enrichment | 16.35 | | -6.15 | |
| **D) LD block 11** | | | | |
| χ^2^ = 299.77, df = 1, p-value < 2.2e-16 | | | | |
|  | | **UTR** | | **S** |
| Expected | | 12622.79 | | 8167.21 |
| Observed | | 13842 | | 6948 |
| % Enrichment | | 9.66 | | -14.93 |
|  | | | | |
| χ^2^ = 13.52, df = 1, p = 2.4e-4 | | | | |
|  | | **NS** | | **S** |
| Expected | | 2592.25 | | 6901.75 |
| Observed | | 2742 | | 6752 |
| % Enrichment | | 5.78 | | -2.17 |

| Table S4. Fits and parameter estimates for demographic models of the 2dSFS of GA and NY, showing the best of five independent runs ranked by AIC. Demographic scenarios included symmetrical migration, secondary contact and no migration (sym_mig, sec_contact, and no_mig, respectively). Each scenario was tested in models allowing for heterogeneity in Ne across the genome due to selection (sym_mig_2N, sec_contact_2N, and no_mig_2N). For scenarios with migration, heterogeneity in migration across the genome (sym_mig_2m, and sec_contact_2m). nu1 and nu2 = Ne of populations 1 and 2 after split (in units of Na), respectively; T = Time of split (2*Na generations); T1 = time between split and secondary contact; T2 = time between secondary contact and present; m = symmetrical migration (2*Na*m); m’ = migration across proportion P of the genome; hrf = Hill-Robertson factor of reduced Ne (nu*hrf) across proportion Q of the genome. | | | | |
| --- | --- | --- | --- | --- |
| Model | LL | AIC | theta | parameters |
| sym_mig_2N | -1587.77 | 3187.54 | 1202.01 | nu1=5.35, nu2=5.80, hrf=0.013, m=2.48, T=0.22, Q=0.12 |
| sec_contact_2N | -1610.39 | 3234.78 | 1135.97 | nu1=5.59, nu2=5.83, hrf=0.019, m=2.52, T1=0.01, T2=0.24, Q=0.12 |
| no_mig_2N | -1850.74 | 3711.48 | 1380.36 | nu1=6.60, nu2=7.46, hrf=0.022, T=0.12, Q=0.064 |
| sec_contact_2m | -2439.44 | 4892.88 | 921.32 | nu1=5.00, nu2=3.38, m=6.31, m’=0.048, T1=0.12, T2=0.30, P=0.12 |
| sym_mig_2m | -2451.58 | 4915.16 | 990.98 | nu1=4.53, nu2=2.78, m=6.29, m’=0.049, T=0.40, P=0.17 |
| sec_contact | -3379.87 | 6769.74 | 1090.52 | nu1=4.69, nu2=4.22, m=1.51, T1=0.15, T2=0.095 |
| sym_mig | -3406.57 | 6821.14 | 1092.98 | nu1=4.56, nu2=4.31, m=1.20, T=0.25 |
| no_mig | -3610.02 | 7226.04 | 1286.52 | nu1=5.06, nu2=5.02, T=0.15 |

| Table S5. Enrichment of Biological Processes Gene Ontology (GO) terms among genes scored by summing the number of OutFLANK outlier SNPs in LD block 8. Terms with FDR-corrected p<0.05 are shown. | | |
| --- | --- | --- |
| GO term | GO ID | p-value |
| positive regulation of humoral immune response | 0002922 | 9.74E-04 |
| formation of animal organ boundary | 0010160 | 1.46E-03 |
| heterotypic cell-cell adhesion | 0034113 | 6.67E-03 |
| cell junction assembly | 0034329 | 7.21E-03 |
| renal filtration | 0097205 | 7.71E-03 |
| dentate gyrus development | 0021542 | 7.76E-03 |
| cell junction organization | 0034330 | 7.80E-03 |
| myoblast fusion | 0007520 | 7.82E-03 |
| skeletal muscle myosin thick filament assembly | 0030241 | 7.82E-03 |
| striated muscle myosin thick filament assembly | 0071688 | 7.82E-03 |
| regulation of transcription from RNA polymerase II promoter in response to oxidative stress | 0043619 | 7.84E-03 |
| substrate-independent telencephalic tangential migration | 0021826 | 7.94E-03 |
| substrate-independent telencephalic tangential interneuron migration | 0021843 | 7.94E-03 |
| formation of anatomical boundary | 0048859 | 8.17E-03 |
| cell-cell junction organization | 0045216 | 9.30E-03 |
| negative regulation of lipid transport | 0032369 | 9.84E-03 |
| syncytium formation by plasma membrane fusion | 0000768 | 0.010 |
| response to glucagon | 0033762 | 0.010 |
| multicellular organismal signaling | 0035637 | 0.016 |
| syncytium formation | 0006949 | 0.016 |
| regulation of AMPA receptor activity | 2000311 | 0.016 |
| bundle of His cell to Purkinje myocyte communication | 0086069 | 0.020 |
| positive regulation of protein processing | 0010954 | 0.021 |
| hemidesmosome assembly | 0031581 | 0.041 |
| carbohydrate derivative transport | 1901264 | 0.043 |
| positive regulation of protein maturation | 1903319 | 0.048 |

| Table S6. Enrichment of Biological Processes Gene Ontology (GO) terms among genes scored by summing the number of OutFLANK outlier SNPs in LD block 18. Terms with FDR-corrected p<0.05 are shown. | | |
| --- | --- | --- |
| GO term | GO ID | p-value |
| positive regulation of RNA splicing | 0033120 | 7.91E-09 |
| positive regulation of cyclic nucleotide metabolic process | 0030801 | 1.38E-05 |
| positive regulation of purine nucleotide metabolic process | 1900544 | 2.92E-05 |
| positive regulation of nucleotide metabolic process | 0045981 | 2.97E-05 |
| presynaptic membrane organization | 0097090 | 3.78E-05 |
| protein localization to synapse | 0035418 | 5.93E-05 |
| presynapse organization | 0099172 | 8.06E-05 |
| positive regulation of cAMP metabolic process | 0030816 | 1.70E-04 |
| heterophilic cell-cell adhesion via plasma membrane cell adhesion molecules | 0007157 | 2.40E-04 |
| regulation of meiotic nuclear division | 0040020 | 4.92E-04 |
| regulation of meiotic cell cycle | 0051445 | 7.34E-04 |
| positive regulation of interleukin-12 production | 0032735 | 1.24E-03 |
| regulation of behavior | 0050795 | 1.49E-03 |
| negative regulation of leukocyte migration | 0002686 | 1.64E-03 |
| regulation of NMDA receptor activity | 2000310 | 3.55E-03 |
| regulation of interleukin-12 production | 0032655 | 4.42E-03 |
| regulation of RNA splicing | 0043484 | 6.22E-03 |
| positive regulation of synapse maturation | 0090129 | 8.56E-03 |
| negative regulation of translational initiation | 0045947 | 0.012 |
| regulation of synapse maturation | 0090128 | 0.017 |
| cartilage morphogenesis | 0060536 | 0.021 |
| regulation of glycoprotein biosynthetic process | 0010559 | 0.021 |
| neurotransmitter-gated ion channel clustering | 0072578 | 0.024 |
| neuron cell-cell adhesion | 0007158 | 0.025 |
| cerebellar granular layer formation | 0021684 | 0.031 |
| cerebellar granule cell differentiation | 0021707 | 0.031 |
| regulation of glycoprotein metabolic process | 1903018 | 0.036 |
| vocalization behavior | 0071625 | 0.046 |
| positive regulation of cGMP biosynthetic process | 0030828 | 0.046 |

| Table S7. Enrichment of Biological Processes Gene Ontology (GO) terms among genes scored by summing the number of OutFLANK outlier SNPs in LD block 24. Terms with FDR-corrected p<0.05 are shown. | | |
| --- | --- | --- |
| GO term | GO ID | p-value |
| regulation of protein localization to plasma membrane | 1903076 | 1.31E-04 |
| regulation of protein localization to cell periphery | 1904375 | 1.35E-04 |
| positive regulation of protein localization to plasma membrane | 1903078 | 1.40E-04 |
| positive regulation of protein localization to cell periphery | 1904377 | 1.40E-04 |
| positive regulation of interleukin-2 secretion | 1900042 | 5.17E-04 |
| positive regulation of protein deacetylation | 0090312 | 5.41E-04 |
| regulation of skeletal muscle contraction | 0014819 | 5.50E-04 |
| cholesterol transport | 0030301 | 6.27E-04 |
| protein-lipid complex subunit organization | 0071825 | 7.09E-04 |
| plasma lipoprotein particle organization | 0071827 | 7.61E-04 |
| regulation of interleukin-2 secretion | 1900040 | 7.78E-04 |
| positive regulation of lipid storage | 0010884 | 1.27E-03 |
| membrane docking | 0022406 | 1.30E-03 |
| maintenance of protein location in nucleus | 0051457 | 1.31E-03 |
| venous blood vessel development | 0060841 | 1.37E-03 |
| sterol transport | 0015918 | 1.99E-03 |
| Sertoli cell development | 0060009 | 6.38E-03 |
| negative regulation of glucose transmembrane transport | 0010829 | 0.010 |
| triglyceride metabolic process | 0006641 | 0.015 |
| mRNA transcription by RNA polymerase II | 0042789 | 0.018 |
| ammonium transmembrane transport | 0072488 | 0.018 |
| positive regulation of epithelial cell migration | 0010634 | 0.019 |
| vesicle docking involved in exocytosis | 0006904 | 0.019 |
| response to retinoic acid | 0032526 | 0.019 |
| positive regulation of p38MAPK cascade | 1900745 | 0.019 |
| nuclear migration | 0007097 | 0.019 |
| maintenance of protein localization in organelle | 0072595 | 0.019 |
| negative regulation of autophagy | 0010507 | 0.020 |
| positive regulation of cell-matrix adhesion | 0001954 | 0.024 |
| nuclear matrix anchoring at nuclear membrane | 0090292 | 0.024 |
| synaptic vesicle exocytosis | 0016079 | 0.024 |
| maintenance of protein location in cell | 0032507 | 0.024 |
| glycerolipid catabolic process | 0046503 | 0.024 |
| cytoskeletal anchoring at nuclear membrane | 0090286 | 0.024 |
| nucleus localization | 0051647 | 0.025 |
| acylglycerol metabolic process | 0006639 | 0.032 |
| lipid catabolic process | 0016042 | 0.032 |
| nuclear matrix organization | 0043578 | 0.032 |
| neutral lipid metabolic process | 0006638 | 0.032 |
| amine transport | 0015837 | 0.033 |
| regulation of cholesterol metabolic process | 0090181 | 0.041 |
| positive regulation of protein localization to membrane | 1905477 | 0.042 |
| Sertoli cell differentiation | 0060008 | 0.042 |
| venous blood vessel morphogenesis | 0048845 | 0.044 |
| regulation of antimicrobial humoral response | 0002759 | 0.045 |
| regulation of protein localization to membrane | 1905475 | 0.046 |
| organic hydroxy compound transport | 0015850 | 0.050 |

| Table S8. Enrichment of Biological Processes Gene Ontology (GO) terms among genes scored by summing the number of OutFLANK outlier SNPs in LD block 11. Terms with FDR-corrected p<0.05 are shown. | | |
| --- | --- | --- |
| GO term | GO ID | p-value |
| peptidyl-lysine trimethylation | 0018023 | 3.96E-09 |
| pollination | 0009856 | 7.91E-09 |
| histone lysine methylation | 0034968 | 1.64E-06 |
| peptidyl-lysine methylation | 0018022 | 8.12E-06 |
| positive regulation of T cell mediated immunity | 0002711 | 8.25E-06 |
| histone methylation | 0016571 | 1.65E-05 |
| positive regulation of Rho protein signal transduction | 0035025 | 7.18E-04 |
| glomerulus morphogenesis | 0072102 | 7.31E-04 |
| regulation of T cell mediated immunity | 0002709 | 7.45E-04 |
| histone H3-K9 methylation | 0051567 | 2.15E-03 |
| lipid particle organization | 0034389 | 3.97E-03 |
| cellular monovalent inorganic anion homeostasis | 0030320 | 6.49E-03 |
| lens fiber cell development | 0070307 | 6.63E-03 |
| cellular anion homeostasis | 0030002 | 9.28E-03 |
| COPII-coated vesicle budding | 0090114 | 9.87E-03 |
| regulation of natural killer cell mediated immunity | 0002715 | 0.03 |
| protein methylation | 0006479 | 0.04 |
| protein alkylation | 0008213 | 0.04 |
| positive regulation of leukocyte mediated cytotoxicity | 0001912 | 0.04 |
| regulation of heart rate by cardiac conduction | 0086091 | 0.04 |
| regulation of ventricular cardiac muscle cell membrane repolarization | 0060307 | 0.04 |
| pericardium development | 0060039 | 0.04 |


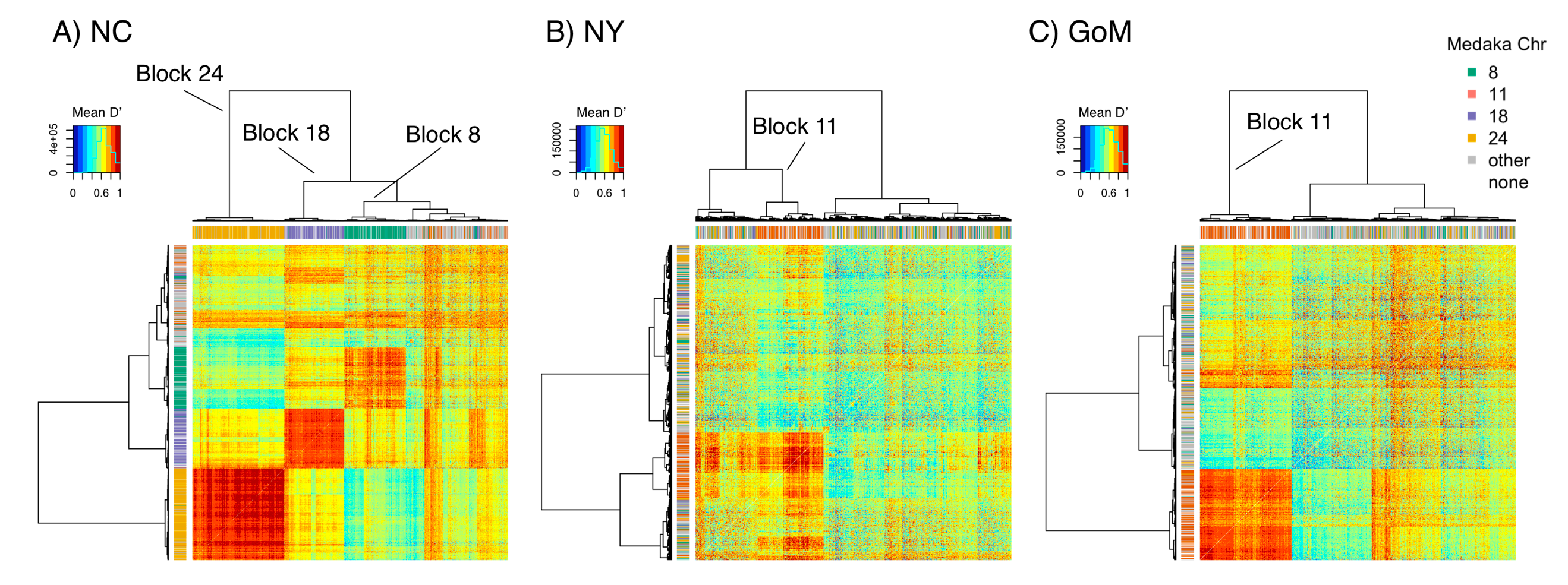


Figure S1. Pairwise linkage disequilibrium (LD) between outlier SNPs on silverside contigs (genes) in **A)** North Carolina (NC), **B)** New York (NY), and **C)** Gulf of Maine (GoM). Pixels are colored by mean D’ across SNPs in pairwise comparisons of all contigs with at least 2 outlier SNPs with MAF>0.1. Dendrograms show the hierarchical clustering of contigs using the symmetrical LD matrix between contig pairs. Row and column panels are colored by which chromosome each contig maps to in the medaka genome. **A)** In NC, the dendrogram shows three clear branches corresponding to three large LD blocks (comprising 391, 255 and 263 contigs, respectively), with a fourth branch grouping 435 contigs with relatively low LD. 91.2% of contigs in LD block 24 that mapped to a medaka chromosome mapped to Chr 24. 82.3% of mappable contigs in LD block 18 mapped to Chr 18, and 89.5% of mappable contigs in LD block 8 corresponded to medaka Chr 8. In GoM, the dendrogram (**C**) formed two branches, one of which (LD block 11) clustered 263 genes with very high LD that predominantly (72.9% of mappable contigs) mapped to medaka Chr 11, whereas the other branch was a cluster of relatively unlinked contigs. The dendrogram in NY (**B**) also shows two clear branches, although many contigs were excluded because of low polymorphism at these contigs in this population. Nonetheless, 184 out of 187 contigs in the high LD block in NY were present in LD block 11 in GoM.


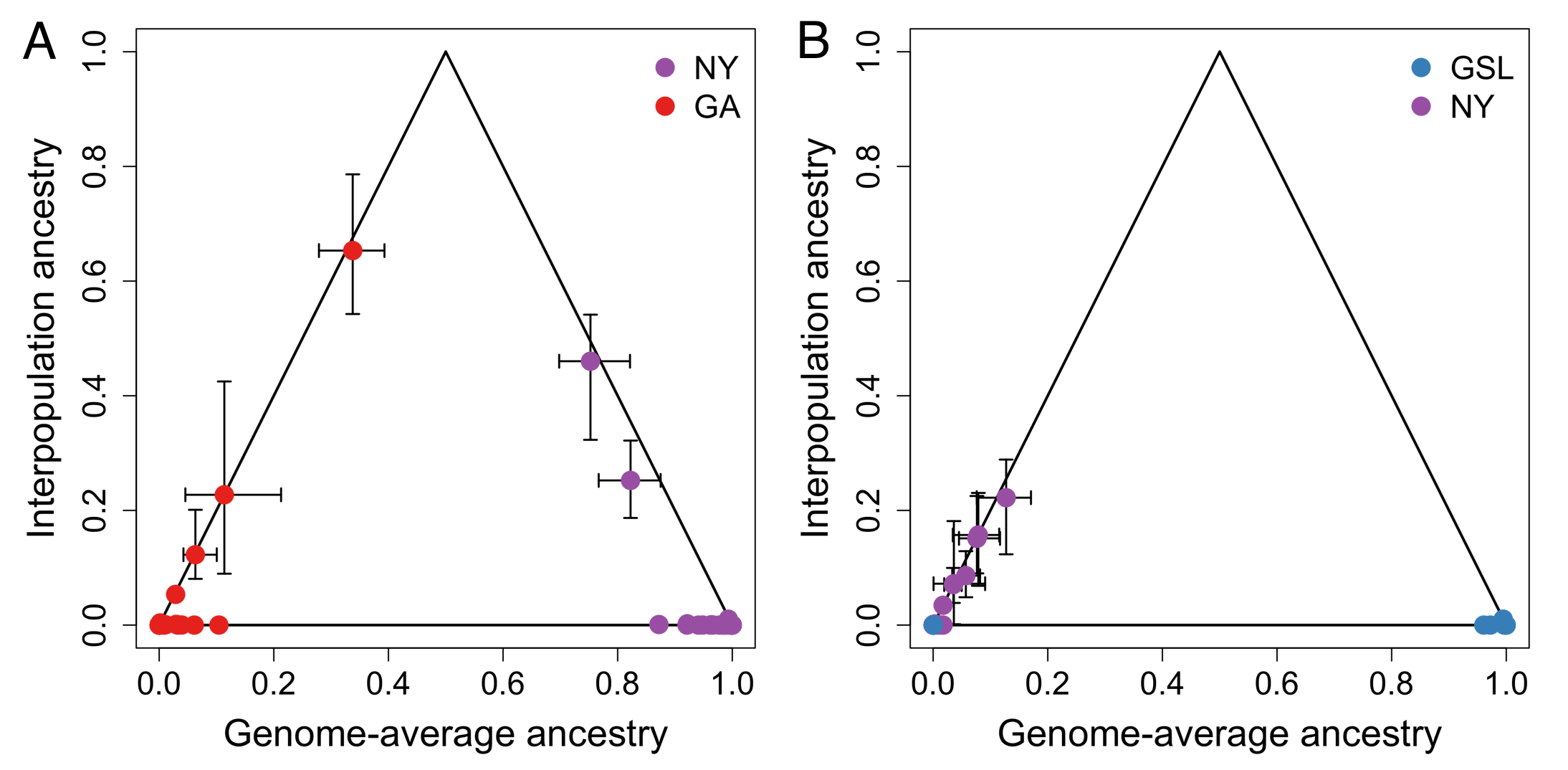


**Figure S2. A)** Interpopulation ancestry analysis of individuals from NY and GA showing high interpopulation ancestry of one individual from GA (the individual that was heterozygous for southern and northern alleles on Chr 8, 18, and 24), and two NY individuals (heterozygous for southern and northern alleles on Chr 24). **B)** One GSL individual shows high posterior assignment to NY, but no interpopulation ancestry. 95% confidence intervals are shown for intervals that do not encompass an interpopulation ancestry of 0. Solid lines indicate the maximum possible inter-source population ancestry for a given global genetic ancestry; individuals on these lines have at least one recent, non-admixed parent.


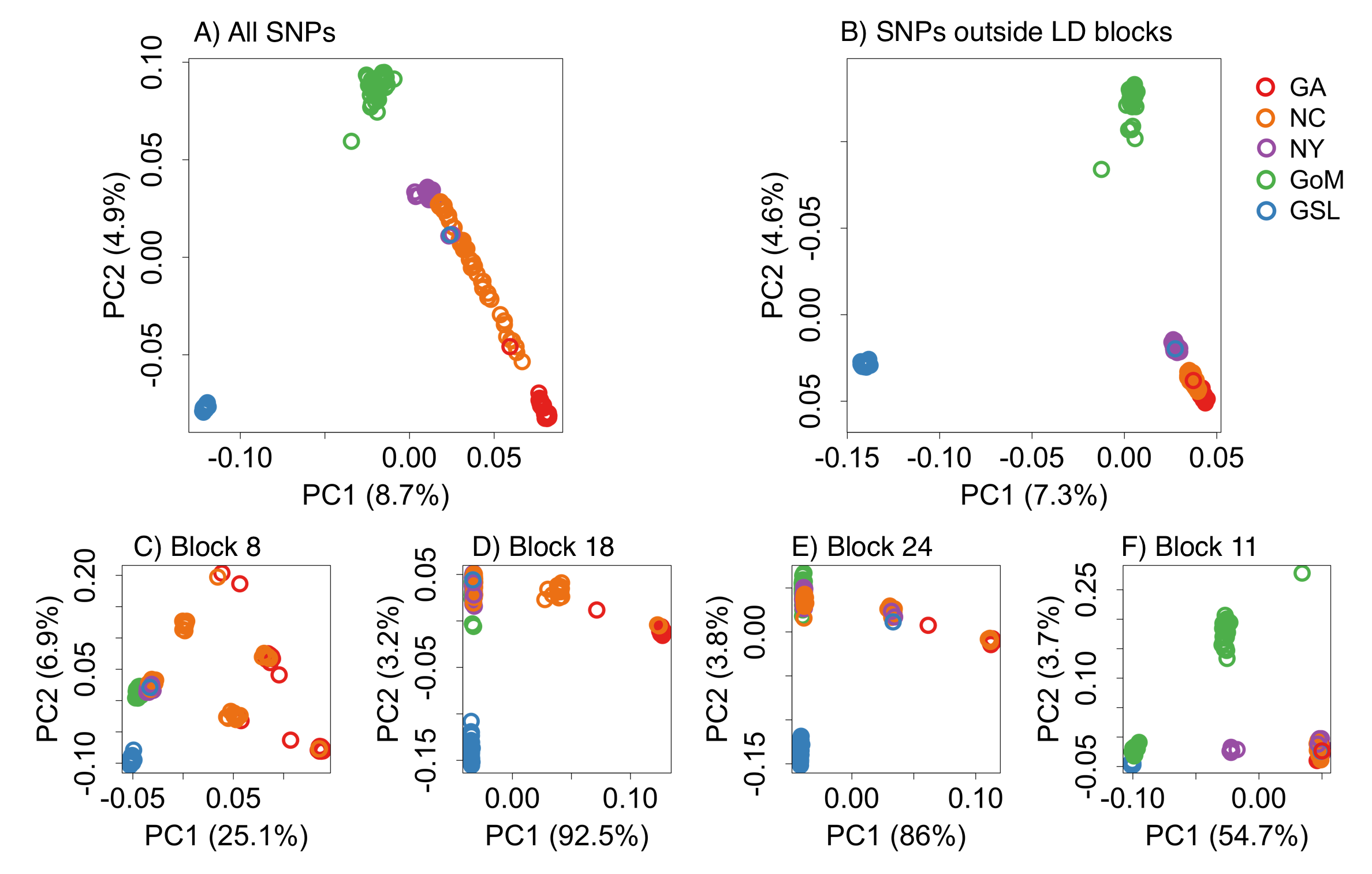


**Figure S3.** Principal components (PC) 1 and 2 using A) all SNPs, B) all SNPs outside of LD blocks, and C-F) SNPs in LD blocks 8, 18, 24, and 11. Note that for Blocks 18, 24, and 11, individuals cluster into three distinct groups on PC1, likely corresponding to the two homozygous and the heterozygous state for each LD block. For Block 8, clustering patterns are more complex suggesting a less preserved haplotype divergence in this block, consistent with the incomplete LD within this block as evident in Fig. 4.


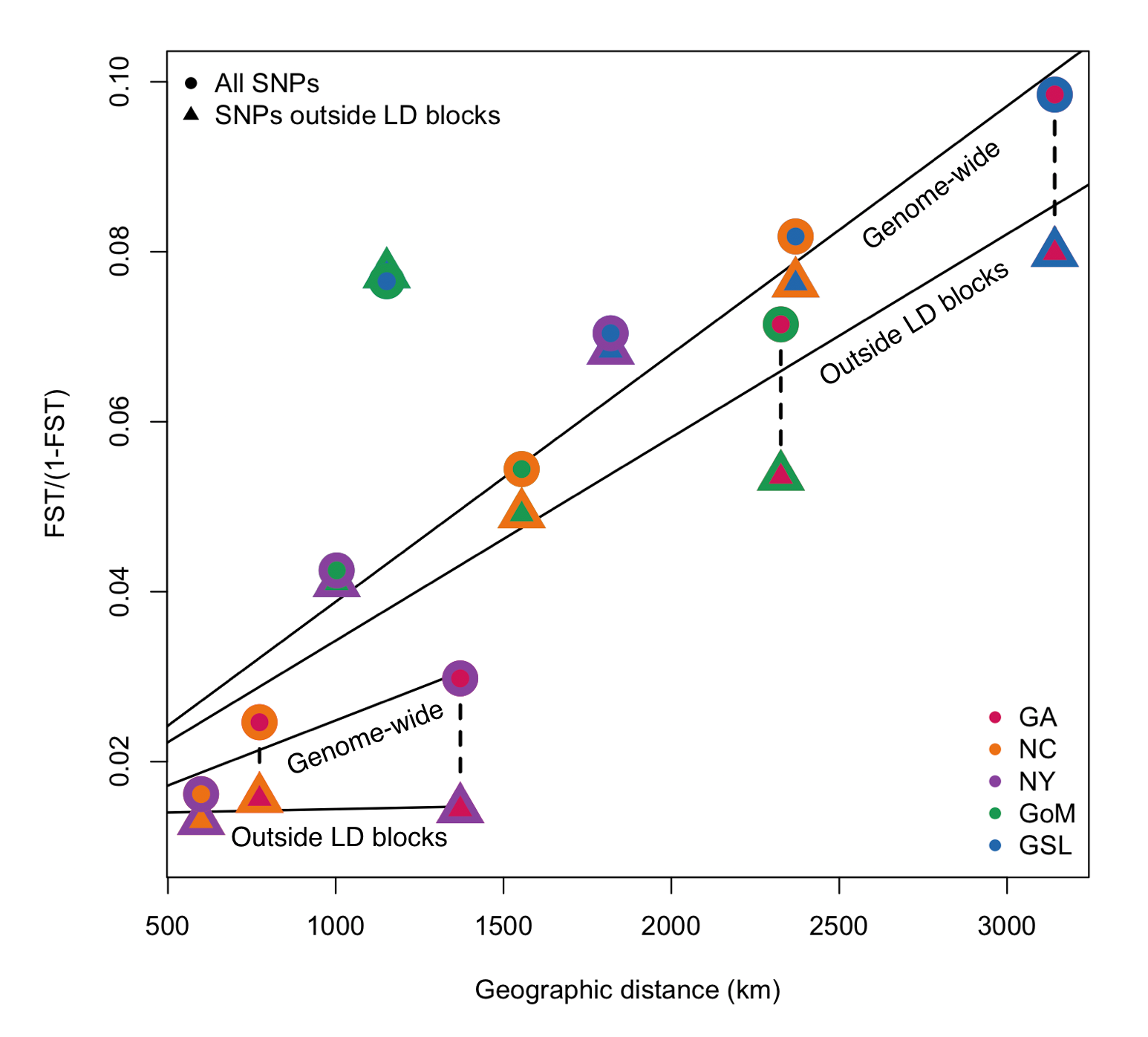


**Figure S4.** Isolation by distance between all pairs of silverside populations, showing differentiation across genome-wide SNPs (circles) and differentiation of SNPs outside LD blocks (triangles). Fitted lines show the slopes for each set of SNPs for all populations, and the shorter, fitted segments show the slopes for the three populations south of Cape Cod only (GA, NC and NY). Points are colored by the pair of populations compared.

**
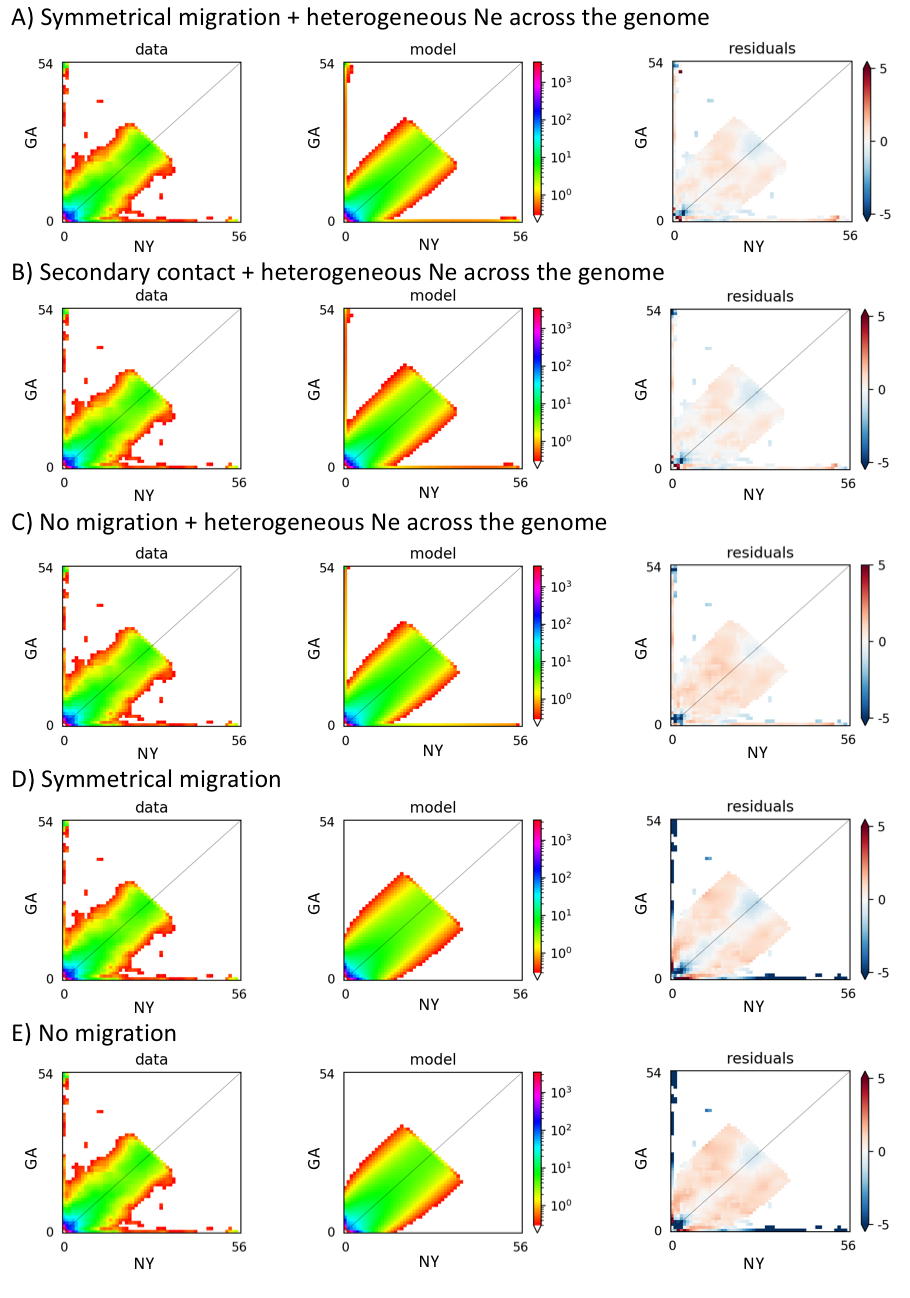
**

**Figure S5.** Fit of demographic models to the joint SFS of silversides from GA and NY, colored by the number of SNPs in each combination of minor alleles in GA and NY. Plots from left to right show the empirical SFS (data), predicted SFS for each model, and residuals between model and data across the SFS. A) Best fitting model of symmetrical migration with heterogeneous Ne across the genome due to selection; B) secondary contact with heterogeneous Ne across the genome; C) no migration with heterogeneous Ne across the genome; D) symmetrical migration; E) no migration.


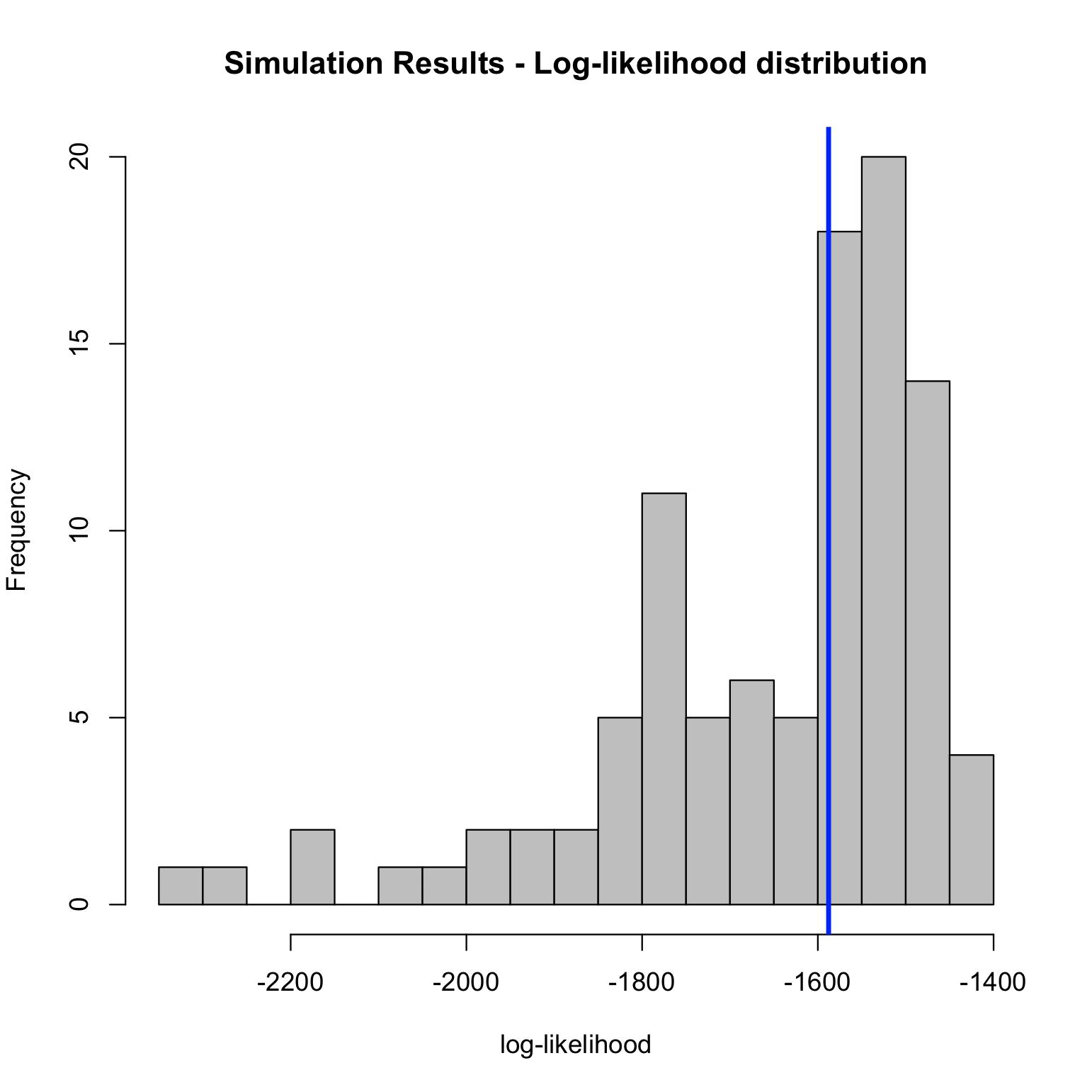


**Figure S6.** Fit of the top-ranked demographic model (symmetrical migration with heterogeneous Ne) to the empirical 2dSFS for GA and NY (blue vertical line), relative to 100 Poisson-sampled 2dSFS simulated under the model (gray bars), showing a good fit of the model to the data.

**References**

Altschul SF, Gish W, Miller W, et al (1990) Basic local alignment search tool. J Mol Biol 215:403–10

Altschul SF, Madden TL, Schäffer AA, et al (1997) Gapped BLAST and PSI-BLAST: a new generation of protein database search programs. Nucleic Acids Res 25:3389–3402

Bolger AM, Lohse M, Usadel B (2014) Trimmomatic: a flexible trimmer for Illumina sequence data. Bioinformatics 30:2114–2120

Conesa A, Götz S, García-Gómez JM, et al (2005) Blast2GO: A universal tool for annotation, visualization and analysis in functional genomics research. Bioinformatics 21:3674–3676

Cunningham F, Amode MR, Barrell D, et al (2015) Ensembl 2015. Nucleic Acids Res 43:D662–D669

Fox EA, Wright AE, Fumagalli M, Vieira FG (2019) ngsLD: evaluating linkage disequilibrium using genotype likelihoods. Bioinformatics

Gillis J, Mistry M, Pavlidis P (2010) Gene function analysis in complex data sets using ermineJ. Nat Protoc 5:1148–1159

Gompert Z, Lucas LK, Buerkle CA, et al (2014) Admixture and the organization of genetic diversity in a butterfly species complex revealed through common and rare genetic variants. Mol Ecol 23:4555–4573

Gutenkunst RN, Hernandez RD, Williamson SH, Bustamante CD (2009) Inferring the joint demographic history of multiple populations from multidimensional SNP frequency data. PLoS Genet.

Jones P, Binns D, Chang H-Y, et al (2014) InterProScan 5: genome-scale protein function classification. Bioinformatics 30:1236–1240

Jouganous J, Long W, Ragsdale AP, Gravel S (2017) Inferring the joint demographic history of multiple populations: beyond the diffusion approximation. Genetics 206:1549–1567

Korneliussen TS, Albrechtsen A, Nielsen R (2014) ANGSD: analysis of next generation sequencing data. BMC Bioinformatics 15:356

Künstner A, Hoffmann M, Fraser BA, et al (2016) The genome of the Trinidadian guppy, Poecilia reticulata, and variation in the Guanapo population. PLoS One 11:

Langmead B, Salzberg SL (2012) Fast gapped-read alignment with Bowtie 2. Nat Methods 9:357

Leaché AD, Portik DM, Rivera D, et al (2019) Exploring rain forest diversification using demographic model testing in the African foam‐nest treefrog Chiromantis rufescens. J Biogeogr 46:2706–2721

Leigh DM, Lischer HEL, Grossen C, Keller LF (2018) Batch effects in a multiyear sequencing study: False biological trends due to changes in read lengths. Mol Ecol Resour 18:778–788

Li H (2011) A statistical framework for SNP calling, mutation discovery, association mapping and population genetical parameter estimation from sequencing data. Bioinformatics 27:2987–2993

Meisner J, Albrechtsen A (2018) Inferring population structure and admixture proportions in low-depth NGS data. Genetics 210:719–731

Murtagh F, Legendre P (2014) Ward’s hierarchical agglomerative clustering method: which algorithms implement Ward’s criterion? J Classif 31:274–295

Myhre S, Tveit H, Mollestad T, Lægreid A (2006) Additional gene ontology structure for improved biological reasoning. Bioinformatics 22:2020–2027

Nielsen R, Paul JS, Albrechtsen A, Song YS (2011) Genotype and SNP calling from next-generation sequencing data

Rougeux C, Bernatchez L, Gagnaire PA (2017) Modeling the multiple facets of speciation-with-gene-flow toward inferring the divergence history of lake whitefish species pairs (Coregonus clupeaformis). Genome Biol. Evol.

Smit A, Hubley R, Green P (2017) RepeatMasker Open-4.0.6 2013-2015 .

Therkildsen NO, Palumbi SR (2017) Practical low‐coverage genomewide sequencing of hundreds of individually barcoded samples for population and evolutionary genomics in nonmodel species. Mol Ecol Resour 17:194–208

Therkildsen NO, Wilder AP, Conover DO, et al (2019) Contrasting genomic shifts underlie parallel phenotypic evolution in response to fishing. Science 365:487–490

Whitlock MC, Lotterhos KE (2015) Reliable Detection of Loci Responsible for Local Adaptation: Inference of a Null Model through Trimming the Distribution of FST. Am Nat 186:S24–S36
